# BicPAMS: software for biological data analysis with pattern-based biclustering

**DOI:** 10.1186/s12859-017-1493-3

**Published:** 2017-02-02

**Authors:** Rui Henriques, Francisco L. Ferreira, Sara C. Madeira

**Affiliations:** INESC-ID and Instituto Superior Técnico, Universidade de Lisboa, Lisboa, Portugal

## Abstract

**Background:**

Biclustering has been largely applied for the unsupervised analysis of biological data, being recognised today as a key technique to discover putative modules in both expression data (subsets of genes correlated in subsets of conditions) and network data (groups of coherently interconnected biological entities). However, given its computational complexity, only recent breakthroughs on pattern-based biclustering enabled efficient searches without the restrictions that state-of-the-art biclustering algorithms place on the structure and homogeneity of biclusters. As a result, pattern-based biclustering provides the unprecedented opportunity to discover non-trivial yet meaningful biological modules with putative functions, whose coherency and tolerance to noise can be tuned and made problem-specific.

**Methods:**

To enable the effective use of pattern-based biclustering by the scientific community, we developed BicPAMS (Biclustering based on PAttern Mining Software), a software that: 1) makes available state-of-the-art pattern-based biclustering algorithms (BicPAM (Henriques and Madeira, Alg Mol Biol 9:27, 2014), BicNET (Henriques and Madeira, Alg Mol Biol 11:23, 2016), BicSPAM (Henriques and Madeira, BMC Bioinforma 15:130, 2014), BiC2PAM (Henriques and Madeira, Alg Mol Biol 11:1–30, 2016), BiP (Henriques and Madeira, IEEE/ACM Trans Comput Biol Bioinforma, 2015), DeBi (Serin and Vingron, AMB 6:1–12, 2011) and BiModule (Okada et al., IPSJ Trans Bioinf 48(SIG5):39–48, 2007)); 2) consistently integrates their dispersed contributions; 3) further explores additional accuracy and efficiency gains; and 4) makes available graphical and application programming interfaces.

**Results:**

Results on both synthetic and real data confirm the relevance of BicPAMS for biological data analysis, highlighting its essential role for the discovery of putative modules with non-trivial yet biologically significant functions from expression and network data.

**Conclusions:**

BicPAMS is the first biclustering tool offering the possibility to: 1) parametrically customize the structure, coherency and quality of biclusters; 2) analyze large-scale biological networks; and 3) tackle the restrictive assumptions placed by state-of-the-art biclustering algorithms. These contributions are shown to be key for an adequate, complete and user-assisted unsupervised analysis of biological data.

**Software:**

BicPAMS and its tutorial available in http://www.bicpams.com.

**Electronic supplementary material:**

The online version of this article (doi:10.1186/s12859-017-1493-3) contains supplementary material, which is available to authorized users.

## Background

The biclustering task has been shown to be essential for improving the status-quo understanding of biological systems, being of particular relevance for expression data analysis (to discover putative transcription modules given by subsets of genes correlated in subsets of conditions [1]) and network data analysis (to unravel functionally coherent nodes [2]). Such relevance is further evidenced by the high number of recent surveys on biclustering algorithms for biological data analysis [3–6]. However, and as an attempt to minimize the complexity of the biclustering task, state-of-the-art biclustering algorithms [1, 7–10] place restrictions on the coherency, quality and structure of biclusters. These restrictions prevent the recovery of complete biclustering solutions and generally lead to the exclusion of non-trivial yet relevant biclusters. Furthermore, state-of-the-art biclustering algorithms generally rely on searches that cannot offer guarantees of optimality [11, 12].

Pattern-based biclustering emerged in recent years as an attempt to address these limitations [13]. Patterns coherently observed on a subset of rows, columns or nodes reveal homogeneous subspaces. In this context, pattern-based biclustering algorithms rely on widely-researched principles for efficiently mining distinct patterns (including frequent itemsets, association rules or sequential patterns) in large databases as the means to identify these subspaces in real-valued matrices or weighted graphs.

The major benefits of pattern-based approaches for biclustering are: *1)* scalable searches with optimality guarantees [11]; *2)* possibility to discover biclusters with parameterizable coherency strength and coherency assumption (including constant, additive, plaid and order-preserving plaid assumptions) [11, 12, 14]; *3)* flexible structures of biclusters (arbitrary positioning of biclusters) and searches (non-fixed number of biclusters) [15, 16]; *4)* robustness to noise and missing values [11] by introducing the possibility to assign multiple symbols or ranges of values to a single data element; *5)* easy extension for labeled data analysis using discriminative patterns [11]; *6)* applicability to sparse matrices and network data [2, 17]; *7)* well-defined statistical tests to assess/enforce the statistical significance of biclusters [18], and *8)* easy incorporation of constraints to guide the search [11].

Furthermore, results on biological data show their unique ability to retrieve non-trivial yet meaningful biclusters with high biological significance [2, 11, 14].

To integrate these dispersed contributions, BicPAMS (**Bic**lustering based on **PA**ttern **M**ining **S**oftware) is proposed to discover biclusters with customizable structure, coherency and quality, yet powerful default behavior. BicPAMS makes available earlier pattern-based biclustering algorithms (including BicPAM [11], BiModule [16] and DeBi [15]), well suited for expression data analysis. Furthermore, BicPAMS implements recent contributions that guarantee the applicability of biclustering towards network data (BicNET [17]), the discovery of order-preserving and plaid models (BicSPAM [12] and BiP [14]) and the incorporation of domain knowledge [19].

This work is organized as follows. The remaining part of this section provides the background on pattern-based biclustering. “Implementation” section describes the behavior of BicPAMS, covering the allowed inputs, parameters and visualization options. “Results” section provides empirical evidence of BicPAMS’ role to unravel non-trivial and relevant putative modules from biological data. Finally, the major implications are highlighted.

### **Definition 1**

Given a real-valued matrix (or network) *A* with a set of rows (or nodes) *X*= {*x*_1_,..,*x*_*n*_}, a set of columns *Y*= {*y*_1_,..,*y*_*m*_} and elements *a*_*ij*_ relating row *x*_*i*_ and column *y*_*j*_ (or relating nodes *x*_*i*_ and *x*_*j*_): the **biclustering** task aims to identify a set of biclusters $\mathcal {B}$= {*B*_1_,..,*B*_*p*_}, where each bicluster *B*_*k*_= (*I*_*k*_,*J*_*k*_) is defined by a subset of rows *I*_*k*_⊂*X* and columns *J*_*k*_⊂*Y* (or two subsets of nodes) satisfying specific criteria of *homogeneity* and statistical *significance*.

**Fig. 1:**
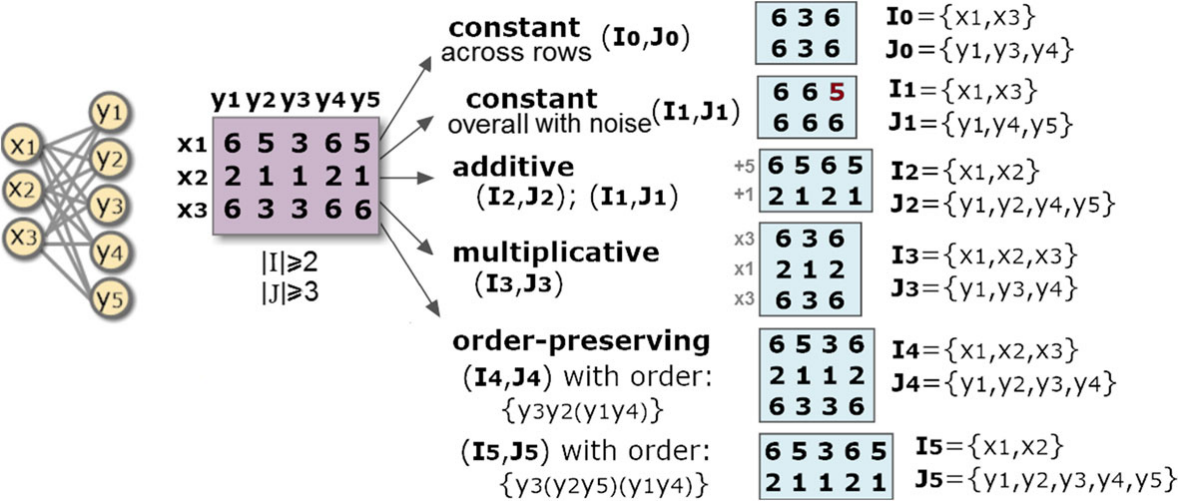
Symbolic pattern-based biclusters with varying coherency assumptions

The placed homogeneity criteria determine the structure, coherency and quality of a biclustering solution, while the statistical significance criteria guarantees that the probability of a bicluster to occur deviates from expectations. The *structure* of a biclustering solution is defined by the number, size, shape and positioning of biclusters. A flexible structure has a non-fixed of arbitrarily positioned biclusters. The *coherency* of a bicluster is determined by the form of correlation among its data elements (coherency assumption) and by the allowed deviations per element against the perfect correlation (coherency strength). The *quality* of a bicluster is defined by the type and degree of tolerated noise. Figure 1 shows biclusters with different coherency assumptions for an illustrative symbolic dataset.

### **Definition 2**

Given a matrix *A*, let the elements in a bicluster *a*_*ij*_∈*B* have **coherency** across rows (patterns on rows) given by *a*_*ij*_=*k*_*j*_+*γ*_*i*_+*η*_*ij*_, where *k*_*j*_ is the value expected for column *y*_*j*_, *γ*_*i*_ is the adjustment for row *x*_*i*_, and *η*_*ij*_ is the noise factor (determining the *quality* of the bicluster). Coherency across columns is identically defined over the transposed matrix, *A* ^*T*^. Let $\bar {A}$ be the amplitude of values in *A*. Given *A*, **coherency strength** is a real value $\delta \in \,[\!0,\bar {A}]$, such that *a*_*ij*_=*k*_*j*_+*γ*_*i*_+*η*_*ij*_ and *η*_*ij*_∈ [ −*δ*/2,*δ*/2].

### **Definition 3**

The properties of *a*_*ij*_ elements define the **coherency assumption**: *constant* when *γ*=0 and *additive* otherwise. *Multiplicative* assumption is observed when *a*_*ij*_ is better described by *k*_*j*_ *γ*_*i*_+*η*_*ij*_. *Symmetries* can be accommodated on rows, *a*_*ij*_ *c*_*i*_ where *c*_*i*_∈{1,- 1}. *Order*-preserving assumption is observed when the values along the subset of columns induce the same linear ordering per row. A *plaid* assumption considers the cumulative effects associated with elementar contributions from multiple biclusters on areas where their columns and rows overlap.

### **Definition 4**

Given a bicluster *B*=(*I*,*J*), the **bicluster pattern** *φ*_*B*_ is the set of expected values (*k*_*j*_) in the absence of noise (*η*_*ij*_=0) and adjustments (*γ*_*i*_=0) according to a fixed ordering of columns: {*k*_*j*_∣*y*_*j*_∈*J*}; while its *support*, |*I*|, is the number of rows satisfying the pattern.

Consider the bicluster (*I*_2_,*J*_2_)=({*x*_1_,*x*_2_},{*y*_1_,*y*_2_,*y*_4_,*y*_5_}) in $\mathbb {N}_{0}^{+}$ from Fig. 1 with an additive coherency assumption across rows. This bicluster can be described by *a*_*ij*_=*k*_*j*_+*γ*_*i*_ with the pattern *φ*={*k*_1_=1,*k*_2_=0,*k*_4_=1,*k*_5_=0}, supported by two rows with additive adjustments *γ*_1_=5 and *γ*_2_=1.

**Pattern-based Biclustering.** The recently exploited synergies between biclustering and pattern mining paved the rise of a new class of algorithms, generally referred as pattern-based biclustering algorithms [13]. Pattern-based biclustering algorithms are natively prepared to efficiently find exhaustive solutions of biclusters and offer the unique possibility to affect their structure, coherency and quality [13]. This behavior justifies the increasing attention paid in recent years to this class of biclustering algorithms by the bioinformatics community for biological data exploration [11, 12, 14–17, 20].

Let $\mathcal {L}$ be a set of items. In the scope of pattern mining research [21], a *pattern* is a frequent composition of items *P*, either an itemset ($P\subseteq \mathcal {L}$), association rule (*P*:*P* ^1^→*P* ^2^ where $P^{1}\subseteq \mathcal {L}\wedge P^{2}\subseteq \mathcal {L}$) or sequence (*P*=*P* ^1^⋯*P* ^*n*^ where $P^{i}\subseteq \mathcal {L}$). Given a set of observations *D*={ *P*_1_,..,*P*_*n*_}, let a *full-pattern* be a pair (*P*,*Φ*_*P*_), where *P* is a pattern and *Φ*_*P*_ is the set of observations in *D* containing *P*. Let a *closed pattern* to be a pattern without supersets with the same support ($\forall _{P'\supset P}|\Phi _{P'}|<|\Phi _{P}|\phantom {\dot {i}\!}$).

Given a real-valued matrix *A*, *pattern-based biclustering* relies on mappings from *A* into *D* and on pattern mining methods able to discover all closed full-patterns, which are used to derive all maximal biclusters satisfying certain coherency (e.g. *η*_*ij*_<*ε*) and structure criteria (e.g. $|\mathcal {B}|>p, |I_{k}|>\theta,(\bigcup _{k} B_{k}\cap A)>\tau $). A maximal bicluster with regards to a specified homogeneity criteria is a bicluster that cannot be extended with additional rows or columns while still satisfying the target criteria. See [22] for a detailed formal view on pattern-based biclustering.

In this context, a pattern-based biclustering solution is *optimal* with regards to certain coherency, quality and structure criteria. The optimality of pattern-based biclustering algorithms is linked with their exhaustive and unrestricted behavior, contrasting with peer greedy and stochastic biclustering algorithms.

The major potentialities of pattern-based biclustering against alternative biclustering approaches include the possibility to: perform efficient searches with guarantees of optimality [12]; discover biclusters with parameterizable coherency assumption and strength [11, 12]; guarantee robustness to noise, missing values and discretization problems through the possibility of assigning or imputing multiple values or symbols to a single data element [11]; discover structures with a non-fixed number and positioning of biclusters possibly characterized by plaid effects [14, 16]; annotate biclusters with a measure of their statistical significance [18]; extend their applicability towards network data and sparse data matrices [2, 17]; and incorporate domain knowledge from user expectations, knowledge repositories and literature in the form of constraints to guarantee a focus on biologically relevant and non-trivial biclusters [22].

**Related work.** Following Madeira and Oliveira’s taxonomy [1], biclustering algorithms can be categorized according to their homogeneity criteria (determined by the underlying merit function) and type of search (defined by whether the merit function is applied within a greedy [7, 23], exhaustive [10, 11] or stochastic [9] algorithmic setting). Hundreds of algorithms were proposed in the last decade to discover biclusters satisfying specific forms of homogeneity, as shown by recent surveys on biclustering algorithms for biological data analysis [3–6]. As a result, some of the algorithms with most visibility have been made publicly available recurring to different software, such as BicAT^1^ [24], biclust^2^ [25], Expander^3^ [10] or BicOverlapper^4^ [26]. However, the available biclustering algorithms (regardless of whether they are provided or not with adequate interfaces) assume very specific forms of homogeneity and therefore do not support the enumerated benefits of pattern-based biclustering approaches. Table 1 synthesizes the inherent properties of the state-of-the-art pattern-based biclustering algorithms and how they tackle the problems of peer biclustering algorithms. Despite their inherent benefits, they are not yet accessible through adequate graphical or application programming interfaces (GUI/API), and their contributions remain dispersed, being the possibility to consistently integrate them still uncertain.

**Table 1:** Recent breakthroughs on pattern-based biclustering: algorithms and tackled limitations

Contribution	Biological output	Behavior	Tackled limitations
*Constant Models* BicPAM [11]	Putative functional modules robust to noise, such as co-expressed genes with a regulatory pattern given by possibly different expression levels across a subset of conditions.	Algorithms consistently combining preprocessing, pattern mining (itemsets and association rules) and postprocessing procedures to guarantee the flexibility and robustness of the outputs.	Flexible structures; Exhaustive (yet efficient) searches; Tolerance to noise; Parameterizable coherence strength.
*Multiplicative and Additive Models* BicPAM [11]	Modules with shifting and scaling factors to deal with the distinct responsiveness of biological entities and handle biases introduced by the applied measurement.	Iterative discovery of pattern differences (shifts) and least common divisors (scales), together with pruning strategies, to learn additive and multiplicative models.	Precise modeling of shifting and scaling factors across rows; Flexible structure and parameterizable quality.
*Order Preserving Model* BicSPAM [12]	Coherent variation of gene expression or molecular concentrations across samples or within a temporal progression (such as stages of a disease or drug response).	Biclustering is parameterized with enhanced sequential pattern miners (by ordering column indexes per row according to the observed values) to flexibly discover noise-tolerant orderings.	Surpasses efficiency and robustness issues of exhaustive peers; Flexible structures with guarantees of optimality, addressing the problems of greedy peers.
*Symmetric* Bic(S)PAM [11, 12]	Modules associated with biological processes simultaneously capturing activation and repression mechanisms within transcriptomic, proteomic or metabolic data.	Combinatorial sign-adjustments (together with pruning principles) to model symmetries and integrate them with scales, shifts and orderings.	Discovery of non-constant biclusters with symmetries; Parameterizable properties.
*Network Modules* BicNET [17]	Coherent modules in homo/heterogeneous biological networks with weighted/labeled interactions. Modules able to capture non-trivial forms of behavior and accommodate less-studied biological entities.	Extension of previous contributions towards biological networks. For this end, new data structures and searches are proposed to effectively and efficiently deal with the inherent sparsity of network data.	Discovery of non-dense modules; Robustness to noisy and missing interactions; Scalable for large networks.
*Plaid Model*BiP [14]	Overlapping regulatory influence in expression data (cumulative effects that multiple biological processes have on a gene at a particular time) and network data (cumulative effects in interactions belonging to multiple modules).	Extended searches to recover excluded areas (due to cumulative contributions on regions where biclusters overlap) and to remove noisy areas. New composition functions and relaxations to deal with noise and non-linear cumulative effects.	Addresses the exact additive plaid assumption with relaxations; No need for all the data elements to follow a plaid assumption; Models non-constant biclusters.
*Constraints* BiC2PAM [19]	Biological modules in accordance with user expectations (e.g. non-trivial homogeneity, satisfying a given pattern or preferred regulatory behavior (such as repression)) or with consistent functional terms.	Extended searches to benefit from background knowledge, including: constraints with succinct, anti-monotone and convertible properties, and incorporation of terms from knowledge repositories.	Focus on regions of interest; Efficiency gains; Removal of uninformative values.

## Implementation

**Fig. 2:**
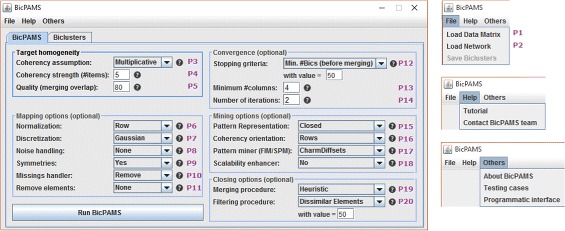
BicPAMS: sound and parameterizable behavior (annotations in *purple*)

**Fig. 3:**
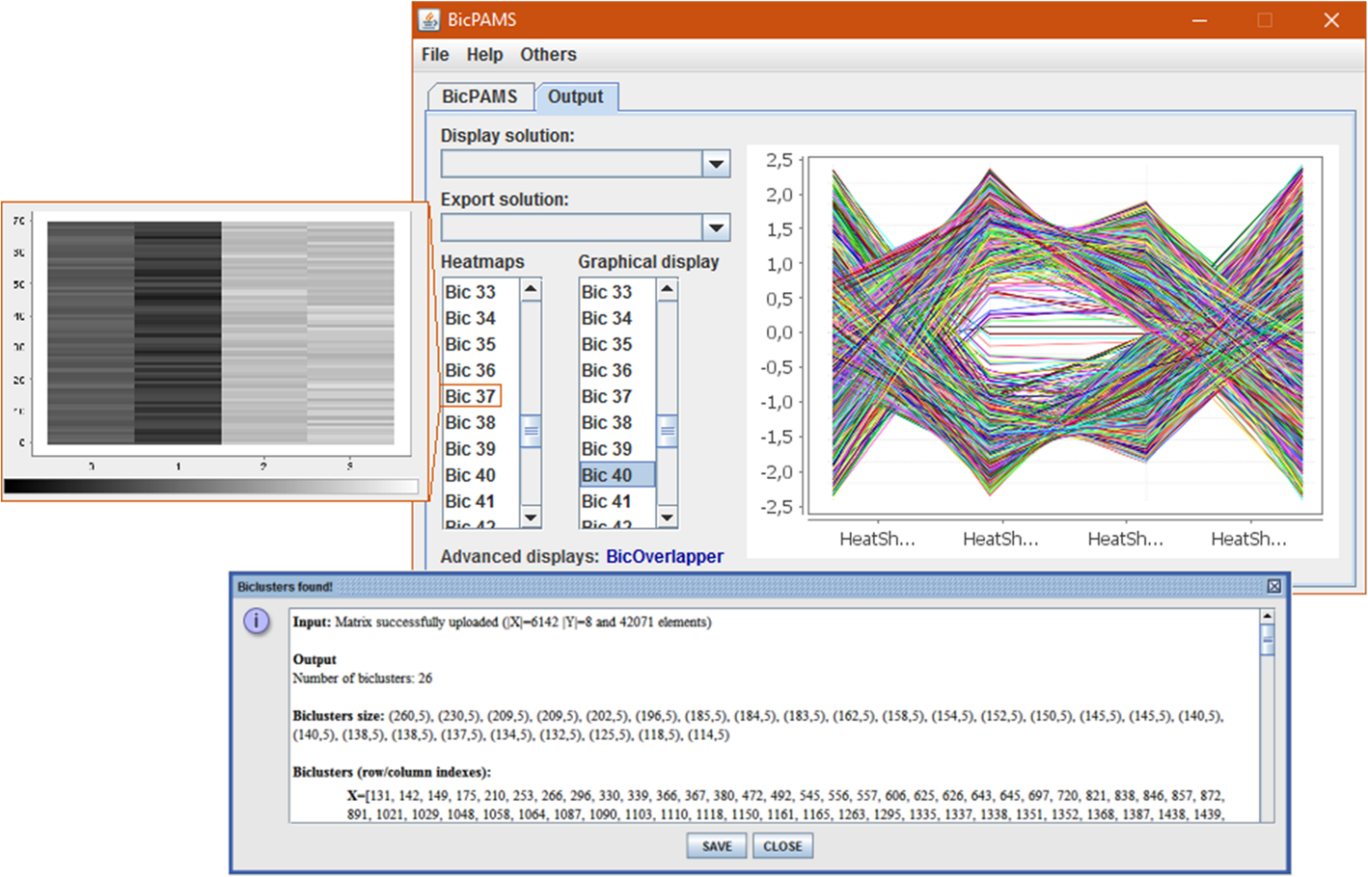
BicPAMS: textual and visual display of results

BicPAMS (Biclustering based on PAttern Mining Software) is the first tool consistently combining state-of-the-art pattern-based biclustering algorithms and making them available within usable interfaces (GUI and API). Figures 2 and 3 provide snapshots of the graphical interface of BicPAMS (where parameters P1 to P20 can be used to determine the desirable properties of the output). First, BicPAMS is described according to the possibilities to parameterize the coherency, structure and quality of its outputs, and the principles to guarantee the efficiency of the underlying searches. We also visit additional contributions of BicPAMS associated with the exploration of potentialities inherent to the integration of pattern-based biclustering algorithms. Second, we cover implementation details associated with the behavior of BicPAMS and the provided interfaces.

### Pattern-based biclustering with BicPAMS

**Coherency of biclusters.** As highlighted in Table 1, BicPAMS allows the search for a parameterizable *coherency assumption* [P3]: constant overall, constant, multiplicative, additive, symmetric or order-preserving. BicPAMS also provides the possibility to robustly select the desirable *coherency strength* *δ* (such that *η*_*ij*_∈[−*δ*/2,*δ*/2]). This is done by fixing the length of the alphabet of discretization $|\mathcal {L}|$ [P4], where $\delta \propto 1/|\mathcal {L}|$. Furthermore, it allows for the inclusion or neglection of symmetries [P9] in order to effectively deal with both symbolic and real-valued datasets with either positive and negative ranges of values or strictly positive ranges of values. Finally, BicPAMS also offers the possibility to select *coherency orientation*: whether verified on rows or columns [P16].

**Structure of biclusters.** BicPAMS relies on the iterative application of dedicated pattern mining searches to guarantee that biclustering can be performed in the presence of a meaningful stopping criteria [P12], such as the minimum number of (dissimilar) biclusters or minimum percentage of the elements in the original dataset covered by the found biclusters. The minimum number of rows [P12] (support) or columns [P13] of biclusters can be optionally inputted to guide the search. Different pattern representations can be used to affect the structure [P15]: simple (all coherent biclusters), closed (all maximal biclusters), and maximal (flattened biclusters with a high number of columns). Furthermore, BicPAMS makes available post-processing options with parameterizable criteria to merge and extend biclusters against the inputted homogeneity criteria and filter biclusters against to prespecified dissimilarity criteria [P19,P20].

**Quality of biclusters.** BicPAMS provides multiple strategies to guarantee robustness to noise. The user can calibrate the desirable level of tolerance to noise through: 1) *post-processing* procedures by specifying the allowed percentage of noisy elements within a bicluster [P5]; and 2) *multi-item assignments* by activating the possibility to assign a parameterizable number of symbols per element based on its original value [P8]. Similarly, BicPAMS guarantees robustness to missing values [P10] by providing imputation methods and enabling the discovery of biclusters with an upper bound on the allowed amount of missing values (particularly relevant when biclustering network data).

**Efficiency.** BicPAMS also relies on enhanced pattern mining searches able to explore efficiency gains from the biclustering task, inputted constraints and desirable structures [P17,P18]. BicPAMS supports frequent itemset mining and association rule mining (including Apriori-based, vertical or dedicated frequent pattern-growth searches [21]), as well as sequential pattern mining (including state-of-the-art and dedicated searches [27]). New searches based on annotated pattern-based trees (F2G search [28]) and diffsets are implemented within BicPAMS to surpass the problems associated with bitset-based searches, as well as searches able to seize efficiency gains from item-indexable properties (IndexSpan [29]). These searches are integrated with heuristics, guaranteeing an effective pruning of the search space in the presence of constraints such as minimum number of columns.

BicPAMS also makes available data structures to deal with sparse data [17] that guarantee a heightened time-and-memory efficiency in the presence of network data. Finally, the application programming interface (API) of BicPAMS can be used to explore additional efficiency gains from non-optimal searches (mining approximate patterns) and the application of pattern mining within distributed/partitioned data settings.

**Synergies.** BicPAMS provides the unprecedented possibility to consistently integrate the previously described options, thus combining the contributions of BicPAM [11], BicNET [17], BicSPAM [12], BiP [14], DeBi [15] and BiModule [16]. Furthermore, BicPAMS can incorporate background knowledge according to the contributions made available in BiC2PAM [19], such as the possibility to remove uninformative elements. The API further supports the specification of constraints and the integrative biclustering analysis of experimental data with annotations derived from knowledge repositories.

In this context, although BicPAMS offers an environment with a substantial number of parameters, it makes available default and dynamic parameterizations that are suitable for the majority of data contexts (see Table 4).

Furthermore, BicPAMS explores efficiency gains from particular combinations of parameters. This is, for instance, the case when BicPAMS is applied with multiple coherency or quality criteria at a time. In this context, the search benefits from new heuristics (based on the principle that biclusters with stricter coherency or quality are contained in biclusters with more flexible coherency or quality) and the joint application of pre- and postprocessing procedures.

### On how to use BicPAMS

**Input and output.** BicPAMS supports the loading of input data according to a wide-variety of tabular and network data formats (see Tables 2 and 3). Upon running BicPAMS, when the stopping criteria is achieved, a success message is displayed, enabling the visualization of the output. Both graphical and textual presentations (heatmaps and signal signatures) of the found biclusters are provided. Biclusters can be filtered, sorted and exported to be stored in knowledge bases or visualized on alternative software.

**Table 2:** BicPAMS: input data, major parameters, and output models

Input: Data	P1 Matrix	The accepted file formats include attribute-relation files (.ARFF) and standard matrix files (such as.TXT). The first line of standard matrix files should specify the column identifiers, while the first entry of each line should specify the row identifier. Tabular data can be either delimited by tabs, spaces or commas.
	P2 Network	BicPAMS accepts any input file format (such as.TXT or.SIG) assuming that: the first line specifies the column identifiers, and each other line specifies an interaction/entry within the network. An entry specifies the nodes and the association strength. Entries can be either delimited by tabs, spaces or commas. In addition to the file, the column index identifying the first node, second node and association strength needs to be inputted. Illustrating, for a network with header “idProteinA,nameProteinA,idProteinB,nameProteinB,weight”, the user should fix (node1,node2,score) indexes as (0,2,4) or (1,3,4). Finally, the user can specify whether each entry is directional from the first node towards the second node or bidirectional. Bidirectional entries increase the density of the network.
Desirable Biclustering Models	P3 Coherency Assumption	The coherency assumption defines the correlation of values within a bicluster. In constant models, an observed pattern (possibly containing different items) is preserved across rows (or columns). In additive or multiplicative models, shifting or scaling factors are allowed per row (or column) in order to allow meaningful variations of the original pattern. In order-preserving models, the values per row induce the same ordering across columns. A plaid model considers the cumulative effect of the contributions from multiple biclusters on areas where their rows and columns overlap. Previous models can further accommodate symmetric factors.
	P4 Coherency Strength	The number of items determines the allowed deviations from expected values. Illustrating, a gene expression matrix parameterized with 5 items will have 2 levels of activation ({1,2}), 2 levels of repression ({-1,-2}) and 1 level of unchanged expression ({0}). By going beyond the differential values, BicPAMS enables the discovery of non-trivial yet coherent and meaningful correlations. To maintain consistency, additive (multiplicative) models should be used with an uneven (even) number of items. When considering order-preserving models, the number of items should be increased to balance the degree of co-occurrences versus precedences.
	P5 Quality	This field specifies the maximum number of allowed noisy/missing elements (determining the minimum overlapping threshold for merging procedures). The tolerance of biclusters to noise can be additionally addressed using noise handlers (see mapping options) and alternative postprocessing procedures.
	P15 Pattern Representation	Closed patterns (default option) enable the discovery of maximal biclusters (biclusters that cannot be extended without the need of removing rows and columns). Maximal patterns gives a preference towards flattened biclusters, possibly neglecting both vertical and smaller biclusters. Finally, the use of simple/all frequent patterns leads to biclustering solutions with a high number of biclusters (possibly contained by another bicluster), which can be useful to guide postprocessing steps. As the user specifies one of these three options, the available pattern miners are dynamically updated.
	P16 Orientation	Coherency can be either observed across rows (default) or columns (searches are applied on the transposed matrix). When the number of columns highly exceeds the number of rows (or vice-versa when searches are applied on the transposed matrix), pattern miners with vertical data formats such as Eclat should be preferred.
	Output	Upon successfully running BicPAMS, a textual and graphical display of the outputs is provided. The user can select the level of details associated with the outputted biclustering solution (statistics only, list of rows and columns per bicluster, disclosure of values per bicluster).

**Table 3:** Additional parameters of BicPAMS along the mapping, mining and closing steps

Mapping Options (includes P4 from Table 2)		P6 Normalization	Depending on the properties of the input data, the user can either normalize data per Row, Column or for the Overall data elements or ignore normalization by selecting the None option. Both outliers and missing values are handled separately.
		P7 Discretization	Real-valued data needs to be discretized to apply pattern-based biclustering (see noise handling to understand how BicPAMs guarantees robustness to discretization drawbacks). The user can select the cut-off points of a Gaussian distribution (default) or fixed ranges of values (equal sized intervals after excluding outliers). Note that fixed ranges can lead to an imbalanced distribution of items. The user can bypass this option for symbolic data by selecting the None option.
		P8 Noise Handler	Multi-item assignments can be considered to handle deviations on the expected values within a bicluster caused by noise or discretization issues. By selecting this option, 2 items are assigned to elements with a value near a boundary of discretization (value in range *c*∈[ *a*,*b*] when min(*b*- *c*,*c*-*a*)/(*b*-*a*) <25%). In this context, a data element becomes associated with a varying number of items, thus increasing the size of data for analysis.
		P9 Symmetries	This option is dynamically selected if the input data is composed by positive and negative values (as it naturally affects the properties of the outputted biclusters). When using symmetric ranges, additive (multiplicative) models should be parameterized with an odd (even) number of items to guarantee consistent shifts (scales).
		P10 Missings Handler	The user can specify what happens in the presence of missing values. Since BicPAMS is natively prepared to analyze sparse data, the Remove option (default) simply signals the algorithms to exclude missings from the searches. Alternatively, the Replace option uses WEKA’s imputation methods to fill missings (the error of imputations can be minimized by simultaneously activating a noise handler). We suggest the use of Remove option for network data and other meaningfully sparse datasets since BicPAMS is able to discover biclusters with missing interactions (see Quality parameter).
		P11 Remove Uninformative Elements	This option supports the possibility to remove uninformative data elements. Zero Entries can be selected to remove the {0}-items, while the Differential option is used to focus on items with high absolute value (e.g. {-3,-2,2,3} when $|\mathcal {L}|$=6). Uninformative elements may correspond to: 1) weak interactions in networks, 2) unchanged expression, 3) healthy evaluations from clinical data, among others.
Mining Options(includes P3, P15 and P16 from Table 2)		P12 Stopping Criteria	The search algorithm runs until any of the available stopping criteria is met. The available options are: 1) minimum number of biclusters before merging (default), 2) minimum covered area by the discovered biclusters (as a percentage of the elements of the input data matrix or network), and 3) minimum support threshold (minimum number of rows per bicluster specified as a fraction of overall rows). The value associated with the selected option should be additionally specified. We suggest the definition of a high number of biclusters (>50) as the default option, in order to guarantee an adequate exploration of the input dataset.
		P13 Minimum *♯*Columns	The minimum number of columns per bicluster can be optionally inputted to promote efficiency and align the outputs according to user expectations. A good principle to fix this value is to use the square root of the number of columns (interactions per nodes) of the input matrix (network).
		P14 *♯*Iterations	BicPAMS default behavior relies on two iterations. For data with large coherent regions that may prevent the discovery of smaller (yet relevant) regions, the number of iterations can be increased to guarantee their discovery. On every new iteration, 25% of the most selected data elements (from the biclusters discovered from the previous iteration) are removed to guarantee a focus on new regions. 3 iterations already guarantee an adequate space exploration for hard data settings.
		P17 Pattern Miner	The available pattern mining algorithms are dynamically provided based on the selected coherency assumption and pattern representation. Sequential pattern miners (SPM) are provided for order-preserving models: PrefixSpan and IndexSpan (an optimized algorithm able to explore gains in efficiency from the item-indexable properties) are made available for simple pattern representations, while BIDE+ is provided for closed pattern representations. Frequent itemset miners (FIM) are selected for the remaining coherency assumptions. AprioriTID, F2G (pattern-growth method for data with a large extent of coherent areas) and Eclat (vertical method for data with a high number of columns) are made available for simple pattern representations. CharmDiffsets, AprioriTID and CharmTID are made available for closed pattern representations, while CharmMFI with diffsets is provided for maximal pattern representations.
		P18 Scalability	This option specifies whether data partitioning principles are applied or not to guarantee the scalability of the searches (only suggested for data with >100 Mb).
Closing Opt.(includes P5)		P19 Merging	Different merging procedures are made available (according to [29]): heuristic (default option) for an efficient quasi-exhaustive merging; and combinatorial and multi-support FIM alternatives for an exhaustive yet more costly postprocessing step.
		P20 Filtering	Filtering is essential to guarantee compact solutions (applied after merging). A biclustered is filtered if it has not enough Dissimilar Elements, Dissimilar Rows or Dissimilar Columns against a larger bicluster. Considering a filtering option with 20% of dissimilar elements. In this context, biclusters sharing more than 80% of their elements against a larger bicluster are removed.

**Fig. 4:**
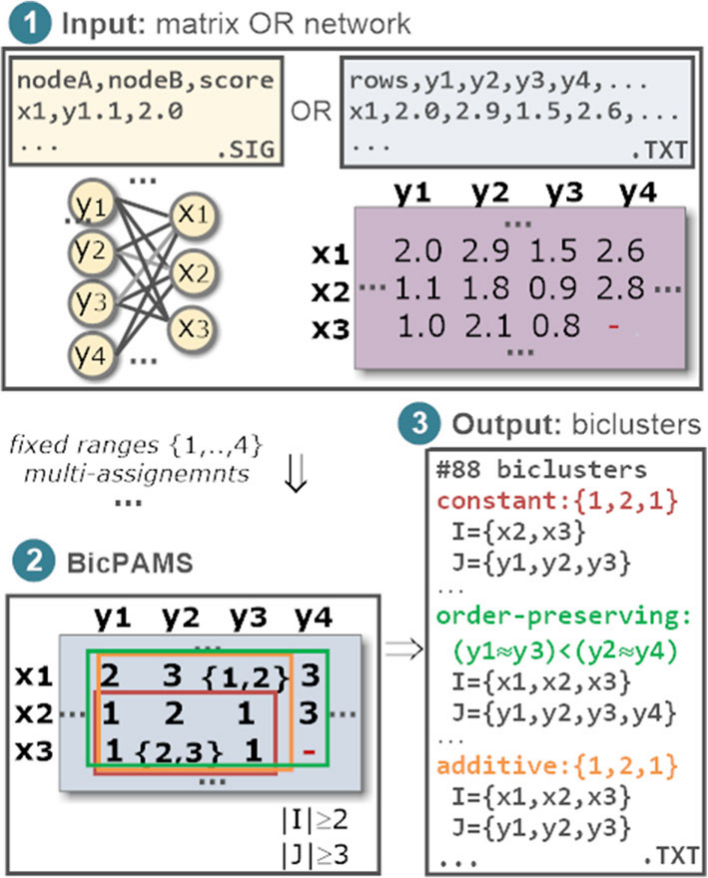
Illustrative application of BicPAMS: input data and output biclusters

Figure 4 provides an illustrative application of BicPAMS for an inputted dataset (either in network or matrix format), showing the outputted biclusters for varying coherency assumptions. For this analysis, we assumed $|\mathcal {L}|=4$, fixed discretization ranges and the assignment of multi-items for an adequate tolerance to noise.

**Graphical interface (GUI).** The desktop interface can be used to soundly parameterize pattern-based biclustering algorithms, as well as to visualize their output. Figures 2 and 3 provide illustrative snapshots. Soundness is guaranteed by: performing automatic form checks, disabling inconsistent fields when specific parameters are selected, and adequately displaying possible causes of errors (such as timeout alerts for heavy requests or data format inconsistencies).

**Console, API and source-code.** Alternatively to the previous interfaces, BicPAMS makes available a console to facilitate its invocation within language-independent scripts, as well as a Java API, the respective source code and the accompanying documentation. The API is essential to: extend the behavior of pattern-based biclustering algorithms for other tasks (such as classification and indexation), and adapt the current behavior to guarantee an optimum ability to handle biological data with specific regularities. Detailed scenarios showing advanced possibilities made available in the API of BicPAMS are provided in the software’s webpage.

**Parameters.** The behavior of BicPAMS can be controlled along its three major stages. First, parameters along the pre-processing stage include: coherency strength (given by the number of items $|\mathcal {L}|$ [P4]), normalization [P6], discretization [P7], imputation [P10], non-informative elements [P11], and the noise range *η*_*ij*_ for multi-item assignments [P8]. Second, parameters along the mining step include: coherency assumption [P3] and orientation [P16], stopping criteria (such as minimum number of dissimilar biclusters) [P13], expectations (such as minimum number of columns) [P14], pattern representation [P15], and algorithmic choice [P17]. BicPAMS supports the parameterization of two post-processing procedures: maximum degree of noisy or missing elements per bicluster (using merging procedures [29]) [P5/P19] and dissimilarity criteria (using filtering procedures [11]) [P20]. The API further provides the possibility to specify a desirable minimum homogeneity threshold to extend or reduce the target biclusters according to a parameterizable merit function [11].

Tables 2 and 3 provide an in-depth description of each of these parameters, showing their default values and how they can be modified according to the properties of the input data and desirable outputs. For an exhaustive exploration of biological data without apriori knowledge of the desirable outputs, BicPAMS can be iteratively applied with varying coherency assumptions, coherency strength ($|\mathcal {L}|\in \{3,4,5\}$) and quality ({60%, 80%, 100%}). Table 4 discusses the default and data-driven parameterizations provided by BicPAMS, showing their adequacy for exploratory yet robust biological data analysis.

**Scalability/limits.** Although biclustering is inherently a computationally complex task, BicPAMS is natively prepared to analyze large-scale matrices/networks (>1 Gb) and, under strict optimality criteria, data with more than one million of entries (∼200 Mb). BicPAMS provides the possibility to select data partitioning procedures. Assuming coherency across rows (patterns on rows), partioning procedures should be applied when (|*X*|>20000∧|*Y*|>*λ*×1000) is satisfied for the constant assumption (*λ*=1) or remaining coherency assumptions (*λ*=0.1). In this context, BicPAMS is able to efficiently analyze expression data with more than 20000 genes (magnitude of human genome) in hundreds of conditions, as well as over sparse biological networks with over 20000 nodes.

**Testing cases.** Synthetic data (resembling biological data), gene expression data and biological networks with >100 Mb are provided with BicPAMS for testing purposes. In BicPAMS webpage, we provide study cases using both synthetic and real data with varying properties to illustrate the multifaceted potentialities of the software.

## Results

Additional file 1 provides extensive experiments that extend the already available assessments of pattern-based biclustering algorithms [2, 11, 12, 14–16] towards: new synthetic and real data, and new performance views (including metrics of completeness, precision and accuracy). In these experiments, the performance of 15 distinct biclustering algorithms was for the first time compared for data contexts with varying size, regularities, and amount and type of noise. The gathered results confirm the enumerated advantages of BicPAMS, including its unique ability to efficiently find exhaustive and flexible solutions with superior robustness to noise.

Follows a brief analysis of some of the results gathered from applying BicPAMS to discover regulatory modules in expression and network data. Additional file 1 extends these analyzes (concerning both the functional enrichment and transcriptional regulation of the discovered modules) and demonstrates the relevance and completeness of pattern-based biclustering outputs against the outputs produced by alternative state-of-the-art biclustering algorithms. The biological relevance of the biclusters was given by the assessment of the over-represented functional terms using an hypergeometric test after Bonferroni correction. We considered a term to be highly enriched if it has a corrected *p*-value below 0.01.

**Case studies on expression data analysis.** Three gene expression datasets were used: *dlbcl* dataset (660 genes, 180 conditions) gathering human responses to chemotherapy [30], *hughes* dataset (6300 genes, 300 conditions) to analyze nucleosome occupancy [31], and *gasch* dataset (6152 genes, 176 conditions) with Yeast responses to environmental stimuli [32]. The goal is to discover coherent expression patterns corresponding to known and putative transcriptional modules associated with the experimental goal (such as elicited immune responses in *dlbcl* and stress responses in *gasch*). Table 5 provides a functional enrichment analysis of pathways, cell lines, transcription factors (TFs) and gene ontology (GO) terms associated with 182 pattern-based biclusters found in the *dlbcl* dataset. BicPAMS was applied with a constant coherency assumption, multi-item assignments, 80% quality, 50% dissimilarity and 3 iterations. The number of genes per bicluster varies between 89 and 166. The enrichment was computed using the Enrichr web tool [33] against terms from the following databases: KEEG, WIKI, Reactome and BioCarta, human PPIs, Gene Ontology, NCI-60 and cancer cell line Encyclopedia, Human Gene Atlas and MSigDB. Each database annotates groups of genes with dedicated terms. Table 5 shows that all the biclusters found are associated with dissimilar sets of coherent terms. The analysis of enriched pathways, TFs, cancer cell lines, target cells, oncogenic signatures and GO terms confirm that the discovered biclusters are associated with meaningful and well-defined putative cellular responses to chemotherapy. Similar analyzes were conducted for the *hughes* and *gasch* data, revealing an identical average number of enriched pathways per bicluster and a significantly higher average number of enriched TFs and GO terms per bicluster.

**Table 4:** Default and dynamic/data-driven parameterizations of BicPAMS

	Parameter	Value	Notes
Major parameters	P3 Coherency assumption	Constant assumption	A default assumption considers a (possibly noise-tolerant) constant pattern on a subset of rows/columns/nodes, providing an adequate degree of flexibility (superior to biclusters with differential/dense values or constant values overall) well suited for initial analyzes.
	P4 Coherency strength	$|\mathcal {L}|$=5 or *δ*=$\bar {A}$/5	Adequate sensitivity to different levels of expression ({-2,-1} {0} and {1,2} sets of symbols correspond to down-regulation, preserved and up-regulation) or association strength. Multiple symbols can be assigned to a single real-valued element to guarantee robustness to noise.
	P5 Quality	80%	Guarantees an adequate tolerance to noise, allowing biclusters to have up to 20% of noisy values.
	P15 Pattern representation	Closed	Closed pattern representations enable the discovery of maximal biclusters (biclusters that cannot be extended without removing rows or columns).
	P16 Orientation	Patterns on rows	In accordance with Def.2. Considering expression data where rows correspond to genes, a bicluster with coherency across rows is defined by a group of genes with the same pattern along a subset of conditions. When rows correspond to conditions, a less-trivial bicluster is given by a group genes with preserved expression spanning a subset of conditions.
Mapping options	P6 Normalization	Row	Normalization of values per biological entity or sample.
	P7 Discretization	Gaussian	Cut-off points of a learned Gaussian curve to minimize imbalanced distributions of items.
	P8 Noise handler	None	By default multi-item assignments are deactivated for an easy interpretation of results. Nevertheless, we suggest the selection of multi-item assignments to guarantee a heightened robustness to discretization drawbacks and noise.
	P9 Symmetries	Dynamic	Symmetries are dynamically selected if the inputted data has negative values. This option can be deactivated to force the biclustering task to not distinguish positive from negative values.
	P10 Missings handler	Remove	Remove is suggested since Quality P5 is already in place to accommodate missing values within biclusters. Nevertheless, Replace option is suggested for data with a considerable amount of missing values.
	P11 Remove uninformative elements	None	By default, no items are removed. Alternative options should be only selected in the presence of knowledge regarding uninformative elements, such as non-differential expression or loose interactions.
Mining options	P12 Stopping criteria	50 biclusters	A minimum number of 50 biclusters (before postpro cessing) is suggested by default since the combination of this option with the quality and dissimilarity criteria leads to a compact set of dissimilar biclusters. This number (as well as the number of iterations) can be increased to guarantee more complete solutions for complex or large datasets.
	P13 Min. *♯*columns	4	Although maximal biclusters have at least 4 columns by default, this number should be increased for datasets where biclusters have a significantly higher number of columns.
	P14 *♯*Iterations	2	Guarantees the removal of small and highly coherent regions in the dataset (after the 1st iteration) to enable the discovery of less-trivial biclusters. This number can be increased to promote a more even distribution of biclusters across the regions of the inputted data.
	P17 Pattern miner	Dynamic	From empirical evidence, CharmDiff is suggested for closed patterns, CharmMFI for maximal patterns, and F2G for simple patterns. When order-preserving coherency is inputted, IndexSpan is suggested by default.
	P18 Scalability	Dynamic	Option activated in the presence of very large datasets (>20 million elements under a constant assumption and >1 million elements for the remaining coherency assumptions).
Closing	P19 Merging	Heuristic	Guarantees an efficient yet quasi-exact postprocessing.
	P20 Filtering	40% dissimilar elements	Guarantees an adequate level of dissimilarity. Biclusters sharing more than 60% of their elements with a larger bicluster are removed.

**Table 5:** Analysis of the highly enriched terms (*p*-value below 0.01 after correction using Enrichr [33]) for the 182 pattern-based biclusters found with BicPAMS in the *dlbcl* dataset (human cellular responses to chemotherapy) against multiple repositories: pathway databases (KEEG, WIKI, Reactome and BioCarta), human PPIs, GO, NCI-60 and cancer cell line Encyclopedia, Human Gene Atlas and MSigDB

	Database	Avg. *♯*terms (*p* <0.01) per bicluster	Summary
Pathways	KEEG Pathways	23±11	Each of the 182 biclusters has a compact set of coherent and significantly enriched pathways in the KEEG database. There is a high dissimilarity (low overlapping) of enriched pathways between biclusters. To illustrate the relevance of enriched pathways to characterize the putative biological role per bicluster, consider the following four discovered biclusters {BK1,BK2,BK3,BK4} with terms showing a corrected *p*-value below 1E-8. BK1 has enriched responses to antigens, including the signaling of FCER1 (controls the production of immune mediators) and NF-kappa pathways. BK2 shows enrichment of more global pathways associated with cancer and immunodeficiency. BK3 has enriched antigen processing and presentation, as well as pathways related with a diversity of autoimmune infections. BK4 is associated with B-cell receptor signaling as expected in chemotherapeutic regulation and pathways regulating the proliferation of (cancerous) cells.
	WIKI Pathways	20±7	Although dissimilarity of WIKI pathways between biclusters is also observed, the overlapping degree of pathways is higher than previous KEEG-based analysis. Consider the highly enriched terms (corrected *p*-value below 1E-8) for three randomly selected biclusters {BW1,BW2,BW3}. BW1 shows enriched signaling pathways associated B-Cell receptor, including signaling of type II interferon, TCR, almost all IL families, chemokine, and TSLP. BW2 has genes closely matching the genes associated with the B-Cell receptor signaling pathway. Finally, BW3 has enriched pathways involved in preventing cell proliferation (as expected after chemotherapy), including G1 to S cell cycle control.
	Reactome Pathways	69±37	The found biclusters have in average a higher number of enriched pathways in the Reactome than in peer databases. Considering two randomly selected biclusters {BR1,BR2} and pathways with enriched *p*-values below 1E-14 after correction. BR1 has enriched pathways associated with immune responses and B-signalings, including cytokine signaling in immune system, interferon signaling, adaptive immune system and immunoregulatory interactions between lymphoid and non-lymphoid cells. BR2 has enriched pathways associated with antigen activation of B-cell receptor and control of cell proliferation (including mitotic G1-G1/S phases, and G1/S and M/G1 transitions).
	BioCarta Pathways	5,5±2,5	The found biclusters are associated with small and dissimilar sets of enriched pathways in the BioCarta database. BioCarta provides unique pathway knowledge, being essential to guarantee a more complete view of the putative roles of biclusters. Let us consider the enriched pathways for 3 randomly selected biclusters, {BW1,BW2,BW3}. BW1 is associated with T-cell receptor (TCR) pathways, including TCR activation by tyrosine kinases, TCR apoptosis and TCR signaling. Similarly to WIKI pathways, BW2 is associated with the signaling of B-cell receptor (BCR) and BW3 with the control of cancerous cell proliferation (inc. regulation of DNA replication and p53 signaling).
Cell lines	NCI-60 Cancer cell lines	5,3±2,1	The majority of biclusters shows a compact set of enriched cell lines – group of genes with unexpectedly high or low expression against remaining cell lines – with few overlapping cell lines between pairs of biclusters. This analysis is key t unravel unique properties of the lymphoma targeted by each bicluster. To illustrate, consider three randomly selected biclusters, {BN1,BN2,BN3}: BN1 was found to be primarily related with follicular lymphoma (RS11846 cell line with corrected 7.9E-9 *p*-value); BN2 was found to be associated with immunoblastic lymphoma (SR cell line with corrected 4.2E-10 *p*-value); and the {MOLT4,SW620,RPMI} cell lines enriched in BN3 (with corrected *p*-values below 1E-8) are associated with T-acute lymphoblastic leukemia, adenocarcinomas and chronic myelogenous leukemia.
	Cancer cell line Encyclopedia	47±30	The majority of enriched cancer cell lines were found to be associated with tumors of the hematopoietic and lymphoid tissues. In general, each bicluster shows an unique set of enriched cell lines. Consider 3 randomly selected biclusters {BC1,BC2,BC3} with enriched cell lines (corrected *p*-value below 1E-10): {DOHH2,KARPAS422,HS611T,WSUDLCL2,HT,SUDHL6} cell lines directly related with diffuse large B-cell lymphoma were enriched in BC1; {MOTN1,ALLSIL,MOLT16} cell lines related with (childhood) T acute lymphoblastic leukemia were enriched in BC2; and {HUT102,EHEB,JVM2} cell lines either pertaining to B-lymphoblastoid or mantle cell lymphoma were enriched in BC3.
Human Gene Atlas		4±1,4	The analysis of terms enriched in the human gene atlas is pertinent to understand the types of cells more likely to be affected by the putative biological responses modeled per bicluster. A few biclusters were found to be associated with effects on the whole blood cells, while the remaining majority of biclusters model more specific biological responses thus showing enrichment on specific types of cells. Considering four randomly selected biclusters {BH1,BH2,BH3,BH4}, we found 721 B lymphoblasts and CD19+ B cells (with *p*-values below 1E-6) associated with BH1, lymphoma burkitts (both Daudi and Raji with *p*-values below 7.2E-4) associated with BH2, CD14+ Monocytes, CD4+ Tcells, CD8+ Tcells (with *p*-values below 1E-4) associated with BH3, and CD33+ Myeloid and D56+ NKCells (with *p*-values below 1E-6) associated with BH4.
MSigDB Oncogenic Signatures		9±1,5	The Molecular Signatures database (MSigDB) tests the enrichment of genes with potential to cause cancer. Interestingly, the majority of the discovered biclusters have a single delineated oncogene (signature with considerably higher enrichment than peer signatures). A few illustrative signatures include: VEGFA UP with V1 DN (8.2E-8) corresponding to genes down-regulated by treatment with angionic factor VEGFA; RPS14 DN with V1 UP (4.3E-11) corresponding to genes up-regulated in CD34+ hematopoietic progenitor cells after knockdown of RPS14; or CAMP UP with V1 UP (3.4E-9) associated with genes up-regulated in primary thyrocyte cultures in response to cAMP signaling. This knowledge further discriminates the putative role of each bicluster.
Regulation	Transcription factors	11±3	Compact and dissimilar sets of TFs were found to be associated with the found biclusters. Illustrating {STAT5A,STAT3,NFKB1}, {AIRE,ESR1,FOXP3,POU5F1,TP53} and {ILF2,CDKN1B,CCND1,UPF1} sets of TFs (with corrected *p*-values below 1E-3) were observed for three randomly selected biclusters. The analysis of TFs is essential to understand the putative regulatory mechanisms modeled by each bicluster. A more detailed analysis of enriched TFs per bicluster is provided in Additional file 1.
	PPI Hub Proteins	83±14	This analysis shows the proteins enriched per bicluster acting as hubs in interaction networks. Despite the large number of enriched hubs per bicluster, it is interesting to notice that biclusters show a low number of overlapping hub-proteins with each other. The analysis of four randomly selected biclusters revealed the {PTPN6,JAK2,CBL}, {GABARAPL1,GABARAPL2, GABARAP}, {SHC1,IL7R,SRC} and {MCC,SLC2A4,CDK1} sets of hub proteins with corrected *p*-values below 1E-10.
Gene Ontology	GO Biological processes	298±90	All biclusters show a high number of functionally coherent terms associated with cellular biological processes. An analysis of the enrichment for some biclusters is provided in Table 6. Complementary analyzes are provided in Table 9.
	GO Cellular component	28±16	The analysis of the enriched cellular components provides complementary information to characterize the putative biological role of each bicluster. Given two randomly selected biclusters from the set of 182 biclusters: one bicluster was associated with cytosol and chromatin-related components (corrected *p*-values below 1E-10), while the other with cell surface and membranes (<1E-10). Unlike biological processes, a few pairs of biclusters share some cellular components.
	GO Molecular function	21±7	Similarly to cellular components, the knowledge of the enriched molecular functions can be used to enlarge the GO-based analysis of biclusters. Each bicluster was found to be associated with a compact set of molecular functions consistently related with the molecular mechanisms underlying immunological responses to chemotherapy. Considering two randomly selected biclusters: the first bicluster showed enriched terms (with corrected *p*-values below 1E-6) associated with antigen binding and the binding of amide, protein complexes and small proteins (inc. chemokine receptor); while the second bicluster showed enriched terms (<1E-6) associated with protein kinase binding and regulation, structure-specific DNA binding, and ATPase activity.

Table 6 lists a compact subset of biological processes with significantly enriched terms for pattern-based biclusters found in *dlbcl*, *hughes* and *gasch* datasets. The analysis of the enriched transcription factors (TFs) of these modules confirms their role in regulating cellular responses to chemotherapy (human) [34] and stress conditions (yeast) [35]. Table 7 further shows the properties of an illustrative subset of pattern-based biclusters with high biological significance as verified by the number of highly enriched terms after Bonferroni correction. These biclusters could not be identified by peer biclustering methods due to the presence of noise-tolerant patterns with multiple expression levels (B1, B2 and B5) and non-constant coherency assumptions (B3, B6, B8). Additional file 1: Tables S6–S9 further stress the relevance of discovering biclusters with plaid, ordering-preserving and symmetric assumptions.

**Table 6:** Illustrative set of terms highly enriched in BicPAMS biclusters

Dataset	ID	Terms	Bicluster with best *p*-value	*♯*Genes in best bicluster
*dlbcl*	Dl1	Translation processes (including translational initiation and elongation)	4.49E-5	81
	Dl2	Transmembrane-related processes (including Golgi apparatus and MHC protein complex)	5.40E-5	83
	Dl3	Defense response; processes related with intra-cellular communication, including receptor activity	4.91-5	162
	Dl4	Innate immune responses, including response to interferon-gamma	1.06E-4	58
	Dl5	Cellular responses to chemical stimulus, including response to cytokine stimulus	0.001	60
	Dl6	Processes targeting the membrane-enclosed lumen associated with the cell cycle process	2.92E-12	81
	Dl7	Immune system processes	1.27E-4	52
*hughes*	H1	Mitochondrion organization and translation; mitochondrial matrix	2.70E-39	416
	H2	Processes concerning the cell periphery and sporulation; cell wall constituent and organization	1.73E-4	370
	H3	Ribonucleoprotein complex biogenesis	3.61E-30	426
	H4	Metabolic and biosynthetic processes of cellular amino acids and carboxylic acids	1.3E-25	581
	H5	Metabolic processes of organonitrogen and sulfur compounds	1.62E-4	504
*gasch*	G1	Cellular response to oxidative stress; generation of precursor metabolites and energy	2.37E-4	296
	G2	Processes to generate precursor metabolites and energy, including the tricarboxylic acid cycle	1.16E-14	954
	G3	Retrotransposon nucleocapsid; viral procapsid maturation	4.34E-6	102
	G4	Processes targeting the intracellular organelle lumen and nuclear lumen	1.17E-47	263
	G5	Nucleolus; ncRNA metabolic processes	1.03E-61	611
	G6	Intracellular non-membrane-bounded organelle; structural molecule activity	5.33E-76	293
	G7	Processes targeting the cytosolic part and, in particular, the ribosomal subunit	1.61E-88	460
	G8	Mitochondrion organization; mitochondrial part; biogenesis of certain protein complexes	2.06E-26	592
	G9	Regulation of macromolecular biosynthetic processes; protein modification	2.28E-13	1019
	G10	Organic substance catabolic and metabolic processes (including carbohydrates)	1.02E-15	648
	G11	General processes associated with ribonucleoprotein complex biogenesis	1.08E-94	784

**Table 7:** Illustrative set of biologically relevant biclusters with different properties

Dataset	ID	Pattern	$|\mathcal {L}|$ (items)	Coherency assumption	Postprocessing	*♯*Genes	*♯*Conds	*♯* *p*−values <0.01	*♯* *p*−values [0.01,0.05]	Best *p*-value
*dlbcl*	B1	FAABFFF	6 (A-F)	constant	Merging with tight overlapping	83	7	41	21	1.97E-10
	B2	AAAABCAA	3 (A-C)	constant	Extensions allowed (tight merging)	153	8	9	1	2.27E-12
	B3	AAAAA/../EEEEE	5 (A-E)	multiplicative	Reducing with high homogeneity	119	5	5	18	4.12E-8
*hughes*	B4	EEECEE	5 (A-E)	constant	Merging allowed	581	6	12	7	1.31E-25
	B5	CCDCBCBCCC	5 (A-E)	constant	Merging with relaxed overlapping	654	10	16	4	1.31E-17
	B6	AAAAAA/...	7 (A-G)	additive	Merging with tight overlapping	476	6	12	10	1.92E-6
*gasch*	B7	AAAGGGA/...	7 (A-G)	multiplicative	Merging with tight overlapping	483	7	57	10	1.24E-81
	B8	AAABACCCAA/...	5 (A-E)	additive	Merging allowed	521	10	17	5	4.57E-12

**Fig. 5:**
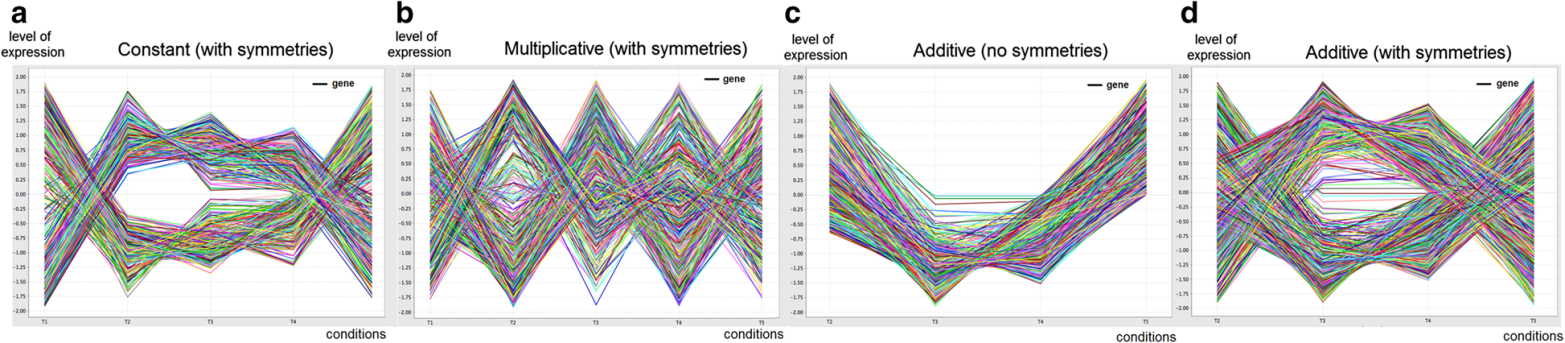
Pattern-based biclusters retrieved from *gasch* data following a constant assumption with symmetries **a**, multiplicative assumption with symmetries (**b**), and additive assumption **c** and **d**

Figure 5 plots four pattern-based biclusters from *gasch* data with distinct coherent responses of genes to heat shock at different points in time. These biclusters rely on constant, multiplicative, additive and symmetric assumptions, each associated with noise-tolerant patterns with five expression levels ($|\mathcal {L}|=5$). Understandably, alternative state-of-the-art biclustering algorithms are not able to discover identical biclusters due to the restrictive assumptions they place on the underlying homogeneity criteria.

**Case studies on network data analysis.** Four biological networks were extracted from DryGIN [36] and STRING v10 [37] databases (Table 8). The goal is to discover putative functional modules given by non-trivial yet coherently interconnected subsets of biological entities. Table 9 illustrates some of all highly enriched biclusters discovered by BicPAMS over the biological networks in Table 8, gathering modules with varying: tolerance to noise (0–15% noisy interactions per bicluster), amount of missing values (0–20% missing interactions per bicluster), coherency assumptions (dense/differential, constant and order-preserving) and coherency strength (*D*_1_- *D*_4_ biclusters with $\mathcal {L}=\{-2,-1,1,2\}, Y_{1}-Y_{4}$ and *H*_1_−*H*_2_ with $\mathcal {L}=\{1,2,3\}, Y_{5}$ and *H*_3_ with $\mathcal {L}=\{1,2,3,4\}$). The biclusters were discovered using multi-item assignments to guarantee their robustness to noise. The results show that all biclusters have highly enriched terms, and the enriched terms per bicluster were also found to be taxonomically related (see Additional file 1). These results further suggest that the found modules are characterized by cohesive putative biological functions. Table 10 characterizes some the enriched pattern-based biclusters, reinforcing the role of BicPAMS to find modules with varying shape, coherency and quality; non-trivial yet biologically meaningful as shown by the number of enriched terms after correction.

**Table 8:** Biological networks used to experimentally assess BicPAMS

Type	Source	Organism	*♯*Nodes	*♯*Interactions	Density	Notes on the weight of interactions
GI (gene interactions)	DryGIN	Yeast	4455	191309	1.0%	Weights (65% negative) from double-mutant arrays [36].
GI (gene interactions)	STRING	Yeast	6314	3759902	1.1%	Known and predicted associations benchmarked from multiple data sources and literature (text mining), and combined through an integrative score [37].
PPI (protein interactions)	STRING	E. Coli	8428	3293416	4.6%	
PPI (protein interactions)	STRING	Human	19247	8548002	2.3%

**Table 9:** Biological role of a subset of BicPAMS’ modules with varying properties

	ID	Homogeneity	*♯*Nodes |*I*|×|*J*|	Putative biological functions: enriched terms (*p*<1E-10)
STRING (yeast)	Y1	dense (high noise-tolerance)	231 ×14	Metabolic processes with incidence on peptide, protein and amide metabolism and biosynthesis.
	Y2	dense (medium noise-tolerance)	217 ×9	Metabolism of nitrogen compounds and other organic substances.
	Y3	constant (few high *a*_*ij*_)	103 ×8	Amino acid activation and tRNA metabolism for aminoacylation.
	Y4	constant (few high or low *a*_*ij*_)	55 ×7	Signal transduction and its related subterms.
	Y5	constant (few high or low *a*_*ij*_)	43 ×6	Phosphorylation terms (with more incidence on protein phosphorylation).
	Y6	order-preserving	176 ×12	Transport of organic acids (incidence on aminoacid transmembrane transport).
	Y7	order-preserving	235 ×9	Oxidation-reduction process and metabolism of aminoacids.
	Y8	order-pres. (few high *a*_*ij*_)	146 ×8	Transport of molecules (highest enrichment found for drug transmembrane).
STRING (human)	H1	dense (high noise-tolerance)	811 ×28	Multiple metabolic processes with incidence on transcription activity.
	H2	constant (few high *a*_*ij*_)	693 ×14	Regulation of intracellular signal transduction (over twenty highly enriched terms).
	H3	constant (few high *a*_*ij*_)	645 ×10	Regulation of molecular functions with incidence on catalytic activity.
	H4	order-preserving	720 ×24	Establishment of protein localization (protein targeting to ER and membrane).
	H5	order-preserving	733 ×29	Protein phosphorylation and its subterms.
DryGIN	D1	dense (high noise-tolerance)	28 ×17	Organelle localization (establishment of spindle and nuclear localization).
	D2	constant (with pos&neg *a*_*ij*_)	22 ×10	Chromatin remodeling and nucleosome organization.
	D3	constant (with pos&neg *a*_*ij*_)	21 ×7	Transport processes for the establishment of protein localization.
	D4	constant (with pos&neg *a*_*ij*_)	19 ×9	Regulation of growth (with incidence on filamentous growth).
	D5	order-preserving	39 ×7	Organelle and nucleous organization.
	D6	order-preserving	54 ×6	Negative and positive regulation of cellular metabolic processes.

**Table 10:** Relevance and exclusivity of BicPAMS’ solutions: properties of some of the found modules in DryGIN

	ID	Type	*♯*Nodes |*I*|×|*J*|	Items	*♯*Enriched terms	Details
DryGIN	G1	constant	18 ×9	{-4,..,-1}	27	Module with coherently strong (–4) and soft (–1) negative interactions.
	G2	symmetric	4 ×9	{-3,..,3}	13	Module with multiple levels of strong (mainly positive) interactions ({ ±3,±2}).
	G3	symmetric	5 ×6	{-2,-1,1,2}	12	Module with either all negative or positive interactions per “row”-node ({ ±1,±2}).
	G4	constant	7 ×5	{1,2}	12	Module with coherent strong (2) and soft (1) positive interactions.
	G5	symmetric	7 ×5	{-2,-1,1,2}	11	Module with either all negative or positive interactions per “row”-node ({ ±1,±2}).
	G6	order	14 ×11	{-3,..,3}	25	Preserved precedences and co-occurrences per “row”-node before postprocessing.
	G7	order	42 ×8	{-2,-1,1,2}	50	Noise-tolerant module with mostly preserved orderings per “row”-node.

## Conclusions

BicPAMS consistently integrates the state-of-the-art contributions from pattern-based biclustering within graphical, scripting and application programming interfaces for the analysis of biological data. BicPAMS is essential for the user-assisted unsupervised exploration of biological data as it overcomes the commonly placed restrictions by peer biclustering algorithms and provides the unprecedented possibility to parameterize the properties of the biclustering solutions. Unprecedentedly, BicPAMS offers the possibility to customize the coherency (including coherency assumption, orientation and strength), quality (including tolerance to noise and missing values), structure and statistical significance (including minimum number of rows and/or columns) of the outputted biclusters. BicPAMS is applicable to dense or sparse, symbolic or real-valued data, and optionally able to incorporate domain knowledge. In order to guarantee the usability of this parametrically rich environment, default parameterizations and simple guidelines (according to the properties of input data and desired output) are provided. BicPAMS further supports multiple data formats and representations of the output, verifying the soundness of requests. Empirical evidence shows that BicPAMS is able to efficiently and effectively discover non-trivial yet coherent biclusters that are robust to noise and biologically significant.

## Availability and requirements

**Project name:** BicPAMS**Project home page:** http://www.bicpams.com **Operating system(s):** All (cross–platform)**Programming language:** Java**Other requirements:** Java v7 or superior**Licence:** GNU General Public License

## Endnotes

^1^ http://www.tik.ee.ethz.ch/sop/bicat/

^2^ http://cran.r-project.org/web/packages/biclust

^3^ http://acgt.cs.tau.ac.il/expander

^4^ http://vis.usal.es/bicoverlapper/

## Additional file

Additional file 1 Supplementary material – experimental assessments of BicPAMS on synthetic and real data accessible at http://www.bicpams.com/appendix. (PDF 685 kb)

## Abbreviations

BicNET: Biclustering networks (algorithm)
BiC2PAM: Biclustering with constraints using pattern mining (algorithm)
BicPAM: Biclustering using pattern mining (algorithm)
BicPAMS: Biclustering based on pattern mining software
BicSPAM: Biclustering using sequential pattern mining (algorithm)
BiModule: Biclustering modules (algorithm)
BiP: Biclustering plaid models (algorithm)
DeBi: Differentially expressed biclustering (algorithm)
DryGIN: Data repository of yeast genetic interactions
GI: Genes interaction
PPI: Protein-protein interactions
STRING: Search tool for the retrieval of interacting genes/proteins
TF: Transcription factor
