# Erratum to: BicPAMS: software for biological data analysis with pattern-based biclustering

**DOI:** 10.1186/s12859-017-1573-4

**Published:** 2017-03-09

**Authors:** Rui Henriques, Francisco L. Ferreira, Sara C. Madeira

**Affiliations:** INESC-ID and Instituto Superior Técnico, Universidade de Lisboa, Lisboa, Portugal

## Erratum

Upon publication of this article [1], it was brought to our attention that the online version of Table 8 contained an error. The correct Table 8 is shown below:

**Table 8:** Biological networks used to experimentally assess BicPAMS

Type	Source	Organism	*♯*Nodes	*♯*Interactions	Density	Notes on the weight of interactions
GI (gene interactions)	DryGIN	Yeast	4455	191309	1.0%	Weights (65% negative) from double-mutant arrays [36].
GI (gene interactions)	STRING	Yeast	6314	3759902	1.1%	Known and predicted associations benchmarked from multiple data sources and literature (text mining), and combined through an integrative score [37].
PPI (protein interactions)	STRING	E. Coli	8428	3293416	4.6%	
PPI (protein interactions)	STRING	Human	19247	8548002	2.3%	

Table 8 has been corrected in the original article.
