# Supplementary material for: BicPAMS: software for biological data analysis with pattern-based biclustering
Source: BMC Bioinformatics. 2017 Feb 2;18:82. doi: 10.1186/s12859-017-1493-3 (PMC5290636; doi:10.1186/s12859-017-1493-3)
Supplement: Supplementary file 1 — Supplementary material – experimental assessments of BicPAMS on synthetic and real data accessible at http://www.bicpams.com/appendix. (PDF 685 kb) [file 12859_2017_1493_MOESM1_ESM.pdf]

Additional file 1: On the Performance of BicPAMS Algorithms

BicPAMS: Software for Biomedical Data Analysis with Pattern-based Biclustering

Rui Henriques, Francisco L. Ferreira, Sara C. Madeira

INESC-ID and CSE Department, Instituto Superior Técnico, University of Lisbon, Portugal
E-mail: {rmch,sara.madeira}@ist.utl.pt

1 Introduction

This document gathers the results and implications from assessing the performance of pattern-based biclustering algorithms within BicPAMS (**Bic**lustering using **PA**ttern **M**ining **S**oftware) on real and synthetic data. The following experiments extend previous results [11–13, 15, 27, 37] in order to offer a more complete view on the capabilities and limitations of BicPAMS. The experiments were performed using an Intel Core i5 2.1GHz with 8GB of RAM.

This document is organized as follows. *Section 2* describes the datasets, biclustering algorithms (and their parameterizations) and performance metrics. The following sections provide the results from assessing the ability of biclustering algorithms to discover biclusters with: varying structure coherency strength (*Section 3*); coherency assumption (*Section 4*); non-trivial plaid effects (*Section 5*); degree of noise (*Section 6*); degree of missing values (*Section 7*). Next, we discuss the biological relevance of pattern-based biclustering solutions learned from expression data (*Section 8*) and biological networks (*Section 9*). Finally, the major contributions of this study are synthesized.

2 Experimental Settings

Synthetic Data. BiGen[1] was used to generate an extensive set of datasets with varying properties for a complete assessment of the strengths and weaknesses of pattern-based biclustering.

First, a high number of matrices were generated by varying the following sets of variables:

- number of rows/columns, number and shape of the planted biclusters according to Table S1;
- coherency strength of the values in the biclusters: either specified as a percentage of the input range of observed values ($\delta \in \{0.05, 0.1, 0.2, 0.4\}$) for real-valued data or as the number of symbols ($|\mathcal{L}| \in \{5, 10, 20\}$) for symbolic data;
- flexible coherency type: planted biclusters following additive, multiplicative, symmetric, and order-preserving models (either one model per dataset or a random combination of distinct models);

[1] https://web.ist.utl.pt/rmch/software/bigen

- plaid structure of biclusters: varying degrees of complexity, where the expected overlapping degree between rows and columns is increased, $\theta \in \{0, 0.1, 0.2, 0.3\}$, according to an additive plaid model with noise ϵ=10% and sets of κ overlapping/interacting biclusters[2] with ϕ distribution of overlaps[3], where $\kappa \in \{2, 3, 5\}$ and $\phi \in \{1, 0.8.0.6, 0.4\}$;

- degree of noisy elements ({0%,2%,5%,10%}) and missing elements ({0%,2%,5%,10%}), and noise factors planted on the a_{ij} values up to $\{0,\frac{1}{2}\delta,\delta,2\delta\}$ of the inputted range of values.

The number of rows and columns for each bicluster follows a Uniform distribution using the ranges in Table S1. By default, we allow for overlapping biclusters according to a plaid structure (10% of overlapping elements) and noise factors (up to ±15% of the range of values), which can make the recovery of the original planted biclusters harder.

For each of these settings we instantiated 40 matrices: 20 real-valued and 20 symbolic. For each set, we varied the generated background values by considering both Uniform and Gaussian distributions, together with the possibility of using symmetries by either allowing or avoiding negative ranges of values. Illustrating for the symbolic case: 10 matrices are generated with background values following a Uniform distribution (5 respecting $U(1,|\mathcal{L}|)$ and 5 respecting $U(-\frac{|\mathcal{L}|}{2},\frac{|\mathcal{L}|}{2})$) and 10 matrices with background values generated according to a Gaussian distribution (5 respecting $N(\frac{|\mathcal{L}|}{2},\frac{|\mathcal{L}|}{6})$ and 5 respecting $N(0,\frac{|\mathcal{L}|}{6})$).

Matrix size (♯rows×♯cols)	100×40	500×70	1000×100	2000×200
♯ hidden biclusters	3	5	10	20
♯ columns in biclusters	[6,8]	[8,10]	[10,12]	[12,15]
♯ rows in biclusters	[14,20]	[25,40]	[40,60]	[60,80]
Area of biclusters	8.9%	4.2%	5.5%	4.7%

Table S1: Properties of the generated set of synthetic datasets.

In addition, we also generated network data with planted modules, respecting the commonly observed topological statistics of biological networks [15]. Variables:

- number of nodes, directionality and density of interactions, and distribution of weights (positive and negative ranges revealing the interaction strength);

- degree of noisy and missing interactions (from 0% to 20%).

- number, size (Uniform distribution on the number of nodes), shape (imbalance on the size of the disjoint sets of each subgraph), overlapping, and coherency (dense, constant, symmetric and plaid) of the planted biclusters:

[2]To illustrate, consider a solution of 20 biclusters, from which subgroups of δ=5 biclusters have interactions with each other but not with biclusters outside the group. When this number matches the total number of biclusters, all processes influence each other forming a complex model of interactions.

[3]ϕ defines the distribution of the overlapping areas between the δ biclusters. ϕ=100% means that the observed plaid effects are a composition of the contributions from all the δ biclusters. ϕ% implies that plaid effects are mainly derived from pairwise overlaps.

	Network nodes (10% density)					Network density (2000 nodes)			
	200	500	1000	2000	10000	1%	5%	10%	25%
♯ Hidden modules	5	10	15	20	30	3	5	10	20
♯ Nodes per module	[20,30]	[30,40]	[40,50]	[50,70]	[100,140]	[50,70]	[50,70]	[50,70]	[50,70]
% Interactions in modules	19,5%	12,2%	7,6%	4,5%	1,1%	22,5%	9,0%	4,5%	2,3%

Real Data. We used three gene expression datasets: *dlblc* dataset (660 genes, 180 conditions) with human responses to chemotherapy [32], *hughes* dataset (6300 genes, 300 conditions) to study nucleosome occupancy [21], and *gasch* dataset[4] (6152 genes, 176 conditions) with Yeast responses to environmental stimuli [9].

In addition, four biological networks were considered: GIs in yeast from DryGIN [18] and STRING v10 [38] databases, and two licensed PPIs in human and E. coli from STRING v10 [38] database. The scores in these networks define the expected strength of influence/physical interaction between genes/proteins:

Type	Organism	♯Nodes	♯Interactions	Density	Notes on the weight of interactions
GI	Yeast	4455	191309	1.0%	Weights (65% negative) from double-mutant arrays [18].
GI	Yeast	6314	3759902	1.1%	Known and predicted associations benchmarked
PPI	E. Coli	8428	3293416	4.6%	from multiple data sources and text mining,
PPI	Human	19247	8548002	2.3%	and combined through an integrative score [38].

Biclustering Algorithms. We selected 16 state-of-the-art biclustering algorithms: 3 approaches able to deliver order-preserving biclusters (OPSM [3], OP-Clustering [22] and BicSPAM [13]), 5 approaches prepared to discover multiplicative and additive biclusters (BicPAM [12], BicNET [15] FABIA with sparse prior Equation [16], ISA [17], Bexpa [30]), 2 approaches able to model plaid structures (BCPlaid [34, 41] and BiP [11]), Cheng and Church (CC) for the discovery of alternative coherency criteria [6], and 5 approaches able to discover biclusters with either constant or differential values (Samba [39], xMotifs [26], and 3 pattern-based biclustering approaches: BiModule [27], DeBi [37] and RAP [28]).

BicPAMS is used to run BicPAM, BicNET, BicSPAM, BiP, BiModule and DeBi. Additionally, we used the following software: R packages fabia[5] and biclust[6] (to run BCPlaid), BicAT [2] (to run OPSM, ISA, CC and xMotifs), (Evo-)Bexpa [30], RAP[7] and Expander[8] (to run SAMBA). The specified number of biclusters for FABIA, Bexpa, ISA, CC and xMotifs (number of starting points) was the number of hidden biclusters plus 10%: $|\mathcal{H}|\times 1.1$. Note that this required specification can be used to guide the search space exploration against other biclustering approaches. Additionally, this specification can optimistically bias Fabia Consensus (FC) levels. The default number of iterations for the OPSM method was varied from 10 to 200 iterations. The pattern-based biclustering methods were parameterized with closed pattern representations, iterative searches with decreasing coherency strength, simple merging option (70% overlap/quality) and filtering of biclusters overlapping with a larger bicluster on more than 70% of its elements. The remaining methods were applied with default parameterizations.

[4]We considered the multiple time points per condition and averaged the replicates of the steady state.
[5]http://www.bioinf.jku.at/software/fabia/fabia.html
[6]http://cran.r-project.org/web/packages/biclust
[7]http://www.mybiosoftware.com/rap-association-analysis-approach-biclustering.html
[8]http://acgt.cs.tau.ac.il/expander

Performance Metrics. The biclustering solutions produced by running the compared algorithms on syntehtic data generated with BiGen were assessed using multiple evaluation criteria. Given the knowledge regarding the planted biclusters, clustering metrics[9], match scores [16, 31] and relative non-intersecting area (RNAI) [4, 29] have been used to understand how well the discovered biclusters, $\mathcal{B} = \{B_1, ..B_k\}$, approximate the planted biclusters, $\mathcal{H} = \{H_1, ..H_p\}$. We selected two effectiveness metrics. First, standard match scores (MS) [31] to assess the similarity of solutions based on the Jaccard index. $MS(\mathcal{B}, \mathcal{H})$ defines the extent to what found biclusters match with hidden biclusters, while $MS(\mathcal{H}, \mathcal{B})$ reflects how well hidden biclusters are recovered (1). To take into account the number of biclusters in both sets, we additionally use the consensus score proposed by Hochreiter et al. [16] (2). We refer to this metric as Fabia Consensus (FC). Let S_1 and S_2 be, respectively, the larger and smaller set of biclusters from $\{\mathcal{B}, \mathcal{H}\}$, and MP be the assigned pairs using the Munkres method based on overlapping areas [25], MC and FC are defined as:

$$\mathbf{MS}(\mathcal{B}, \mathcal{H}) = \frac{1}{|\mathcal{B}|} \Sigma_{(I_1,J_1)\in\mathcal{B}} max_{(I_2,J_2)\in\mathcal{H}} \frac{|I_1 \cap I_2|}{|I_1 \cup I_2|}, \tag{1}$$

$$\mathbf{FC}(\mathcal{B}, \mathcal{H}) = \frac{1}{|\mathcal{S}_1|} \Sigma_{((I_1,J_1)\in\mathcal{S}_1,(I_2,J_2)\in\mathcal{S}_2)\in MP} \frac{|I_1 \cap I_2| \times |J_1 \cap J_2|}{|I_1| \times |J_1| + |I_2| \times |J_2| - |I_1 \cap I_2| \times |J_1 \cap J_2|}. \tag{2}$$

3 Discovery of Biclusters with Varying Size and Coherency Strength

Figure S2 compares the performance of BicPAMS algorithms for the analysis of the synthetic data settings described in Table S1, assuming fixed coherency strength ($\delta = \frac{1}{5}$ for real-valued data and $|\mathcal{L}|$=5 symbols for symbolic data), and post-imputation of noise on the generated values (up to 15% of the input range of values). For this coherency strength, results show the important role of using exhaustive searches able to find biclusters with non-differential coherencies. This explains the better performance of pattern-based biclustering approaches in terms of MS($\mathcal{H}, \mathcal{B}$) score (completeness/coverage) and also MS($\mathcal{H}, \mathcal{B}$) score (correctness/precision). The exhaustive nature of BicPAMS' searches and its ability to rely on multiple discretization levels without risk of introducing noise (by assignment multiple items for values near ranges-boundaries) explains its better performance. The performance of peer pattern-based methods is penalized when noise handlers and merging procedures are not consider. This results in the exclusion of rows (due to the inability to model elements a_{ij} participating in overlapping regions or with a high degree of planted noise) as well as in the partitioning of biclusters (leading to a high number of smaller biclusters), downgrading the FC levels. Although the gains on the effectiveness of pattern-based methods come at a cost on their efficiency, they are still able to perform exhaustive searches in useful time for computationally complex settings. As FABIA, ISA and OPSM are primarily tuned to discover non-constant coherencies, high degrees of noise are associated with the discovered biclusters, penalizing their matching scores. Illustrating, the FC levels of OPSM are strongly penalized since OPSM tends to output

[9]Clustering metrics measure the ability to correctly group rows (or columns), that is, of attaining high intra-cluster similarity and low inter-cluster similarity. Entropy and F-measure metrics are the common choice [1,36]. F-measure can be further decomposed in terms of recall (coverage of found rows by a hidden cluster) and precision (absence of rows present in other hidden clusters).

a large number of biclusters with distinct shapes than the planted ones. Although all approaches are scalable for medium-sized matrices, efficiency deterioration is faster for OPSM, BicPAM and CC.

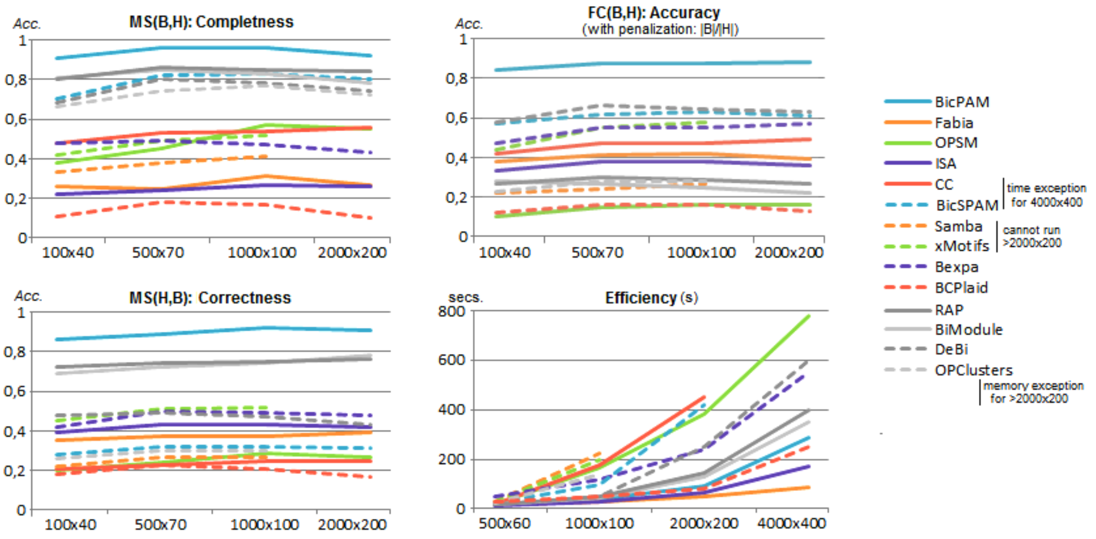

Figure S1: Comparing the performance of state-of-the-art biclustering approaches on data settings with varying properties (Table S1) assuming constant coherencies with fixed strength.

Coherency Strength. Figure S2 assesses the ability of pattern-based biclustering algorithms to discover planted biclusters with varying coherency strength (ranging from δ=0.1 to δ=0.5). With an increasing loose coherency strength, the probability of background values to form a non-planted bicluster is higher. For this reason, we introduced a weighting criteria ν to affect the average number of rows and columns, where ν=1 for $\delta=\frac{1}{5}$, ν=0.8 for $\delta=\frac{1}{10}$, ν=1.2 for $\delta=\frac{1}{4}$, ν=1.5 for $\delta=\frac{1}{3}$ and ν=2 for $\delta=\frac{1}{2}$. This is easily accomplished with BiGen by affecting the Uniform distribution of rows and columns with $U(\nu\times\text{a},\nu\times\text{b})$. Again, this analysis also confirms the superiority of pattern-based biclustering algorithms available in BicPAMS to guarantee noise robustness are employed. Understandably, the biclustering approaches better prepared to discover differential coherencies, such as SAMBA, perform better with a loose coherency strength. Similarly, approaches that tend to discover biclusters with arbitrary-high levels of noise, such as CC, are also less prone to errors when considering loose coherency strength. Contrasting with these sets of approaches, the performance of the remaining approaches is either preserved or even improved with an increased coherency strength. Understandability, a loose coherency strength (corresponding to a smaller number of symbols in symbolic settings) turns the matrix denser, decreasing the efficiency bounds of the analyzed biclustering methods.

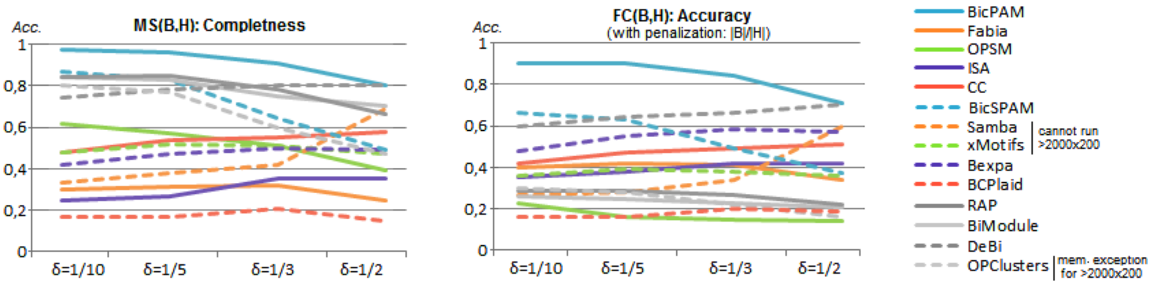

Figure S2: Performance of biclustering approaches on data with planted constant biclusters with varying coherency strength.

4 Performance in the presence of Biclusters with Non-Constant Coherencies

In order to assess the ability of BicPAMS algorithms to recover planted biclusters with non-constant coherencies, we maintained part of the previous experimental data settings and used BiGen to vary the type of coherency of the planted biclusters. Additionally, we considered size adjustments on the planted biclusters to guarantee their significance. The number of rows and columns for additive, multiplicative and order-preserving biclusters are, respectively, 1.2, 1.5 a 2 times the size of constant biclusters provided in Table S1. Figures S3, S4 and S5 illustrate the performance of the compared methods for datasets with additive, multiplicative and order-preserving regularities and fixed coherency strength (δ=0.2). In order to promote the readability of these charts, we excluded the performance of the approaches not prepared to discover biclusters under these assumptions. Results from these figures show the superior performance of BicPAM, for the exhaustive discovery of additive and multiplicative biclusters, and BicSPAM, for the exhaustive discovery of order-preserving biclusters. Their superiority is shown both in terms of MS($\mathcal{B},\mathcal{H}$), that is, the majority of the discovered biclusters are well described by the hidden biclusters (correctness), and MS($\mathcal{H},\mathcal{B}$), that is, the majority of the hidden biclusters can be mapped into a discovered bicluster (completeness). Yet, these levels are slightly worse than the comparable levels for the discovery of constant coherencies due to the higher probability of background values to form a non-planted additive, multiplicative and (in particular) order-preserving bicluster. Again these gains come with a cost in efficiency. In particular, the search for order-preserving models is the most computationally expensive option followed by the search for additive models. The multiplicative model is more efficient since it relies on data transformations associated with sparser matrices. Fabia is a competitive option for the discovery of additive and multiplicative biclusters. However, it is not prepared to deal with overlapping areas and accommodates high levels of noise since it not able to approximate the parameterized coherency strength, leading to biclusters with a larger number of false positive rows. Although ISA is tunned to discover biclusters with gradual changes on values, its scoring schema based on self-consistency property is not well suited to discover biclusters with delineated additive and multiplicative factors. Although OPSM and OP-Clustering can be used to discover biclusters with additive and multiplicative factors, the discovered biclusters include many non-planted rows and columns as they seek to model order-preserving coherencies. Contrasting, OPSM and OP-Clustering are well prepared to recover planted order-preserving

biclusters. Still, OPSM is not able to recover some of the planted biclusters and OP-Clustering suffers from memory problems for the larger data settings. For small datasets, the performance of OP-Clustering is slightly inferior than that of BicSPAM due to the absence of noise-handling options.

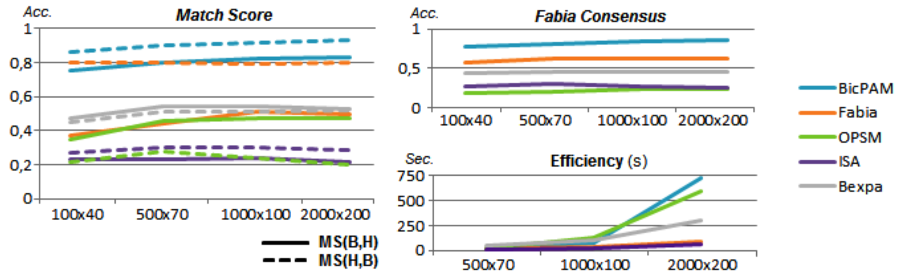

Figure S3: Comparing the performance of biclustering approaches for the recovery of planted biclusters with additive coherency.

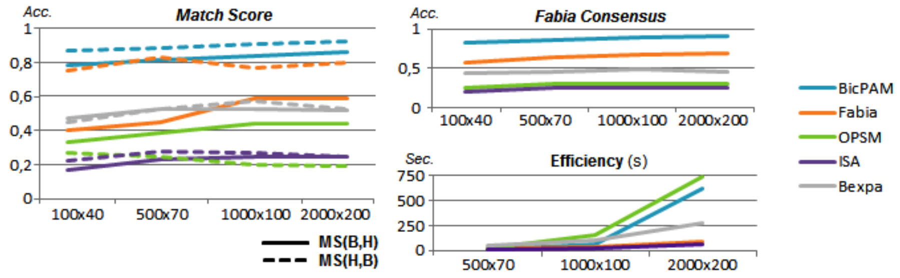

Figure S4: Comparing the performance of biclustering approaches for the recovery of planted biclusters with multiplicative coherency.

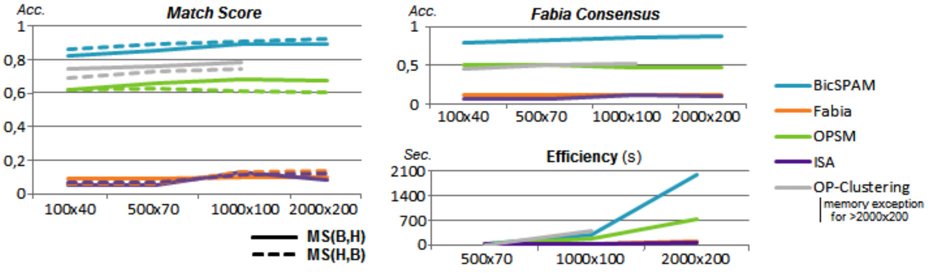

Figure S5: Comparing the performance of biclustering approaches for the recovery of planted biclusters with order-preserving coherency.

5 Performance in the presence of Plaid Structures

Only few biclustering algorithms are prepared to recover meaningful regions in the planted biclusters where overlaps occur. Thus, their performance linearly degrades with an increase in the degree of overlapping between biclusters. Yet, a few biclustering methods are prepared to model arbitrary-high degrees

of overlapping between biclusters based on the plaid model. For this purpose, we planted plaid structures with varying degree and complexity of interactions between biclusters. We considered BiP (using BicPAMS) and BCPlaid[10] biclustering algorithms, well positioned to discover biclusters according to a plaid model. We compare the performance of these methods with an exhaustive non-plaid solution given by the set of maximal biclusters with varying coherence strength (using BicPAM with default postprocessing options). Using the default 1000×100 setting, we varied two parameters for this assessment. First, we controlled the overlapping degree θ to affect the extent of plaid effects. Second, we varied the distribution of overlapped areas ϕ among the κ biclusters to affect the complexity of the plaid model for a fixed number of interacting biclusters κ=5 (two groups of 5 biclusters). Figure S6 provides the match scores for these settings. We can observe that the differences in performance between BiP and biclustering methods not prepared to deal with plaid models increase for high degrees of overlapping due to the extent of plaid effects. BiP outperforms BCPlaid due to the exhaustive nature of its searches and the fact that it does not impose that all the observations (elements in the dataset) fit the plaid model. In this context, since BCPlaid imposes a modular decomposition of the overall data according to a set of biclusters, it is not an appropriate option for the generated plaid structures. We also observe that the adequate identification of plaid effects (BiP with iterative adjustments) becomes essential to deal with complex plaid effects.

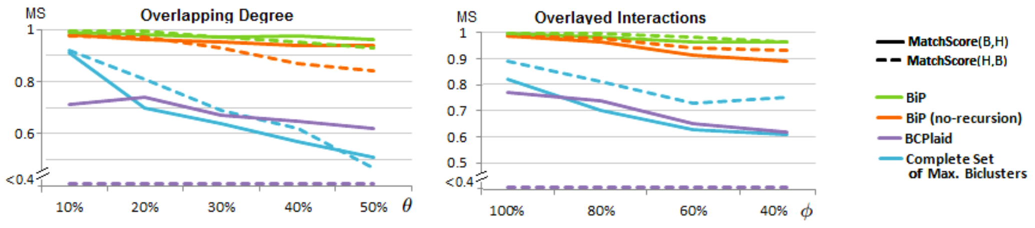

Figure S6: Assessing the ability of biclustering approaches able to model plaid effects to recover biclusters from plaid structures with varying overlapping θ-*extent* and ϕ-*complexity*.

6 Performance in the presence of Noisy and Missing Data

Noisy Data. In order to test the robustness of pattern-based biclustering methods, we generated data with: *1)* parameterizable percentage of noisy elements, and *2)* deviations on the generated values according to different distributions. For this analysis, illustrated in Figure S7, we considered the 1000×100 data setting with: *1)* a varying percentage of noisy elements (from 0 to 10%), and *2)* a varying degree of deviations on the background values given by Uniform distributions ranging from $U(-\frac{1}{2}\delta,\frac{1}{2}\delta)$ to $U(-2\delta,2\delta)$. Generally, we can observe that, although biclustering approaches are able to deal with small portions of noise, their performance naturally degrades with an increasing level of noise. In pattern-based biclustering methods (BicPAM, BicSPAM, BiModule, RAP, DeBi), the penalization is greater for planted deviations on overall values than for planted noisy elements since these methods rely on postprocessing options

[10]BCPlaid is an improved implementation of the original plaid models [19] provided by Turner et. al [41] and implemented by R. Santamaria [34]. Similarly to other existing plaid models [5, 10, 35], BCPlaid aims to learn the model by minimizing the error associated with the plaid equation [5].

well-prepared to recover noisy elements. Contrasting, the performance of the remaining biclustering methods tend to be more affected by the presence of elements with arbitrary-high levels of noise in the biclusters, and less penalized by deviations on the values since these deviations have a less impact on the homogeneity of the biclusters.

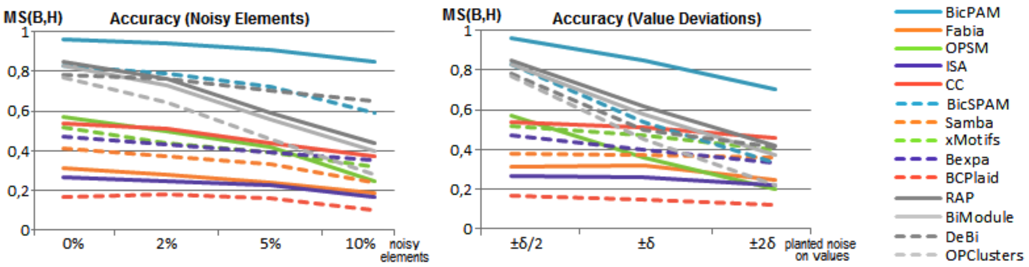

Figure S7: Performance of biclustering approaches over data with varying number of noisy elements and degree of deviations on the values from the expected coherency.

The planted noise can be used to tune the behavior of pattern-based biclustering methods. Figure S8 evaluates the impact of using adequate postprocessing strategies over pattern-based biclustering solutions in order to deal with settings with medium-to-high degrees of planted noisy elements. In this context, we observe that the: post-extension of biclusters (Figure S8a), merging of biclusters with parameterizable degree of overlapping (Figure S8b) and removal of rows/columns of biclusters with parameterizable degree of noise (Figure S8c) are critical to maintain attractive levels of accuracy.

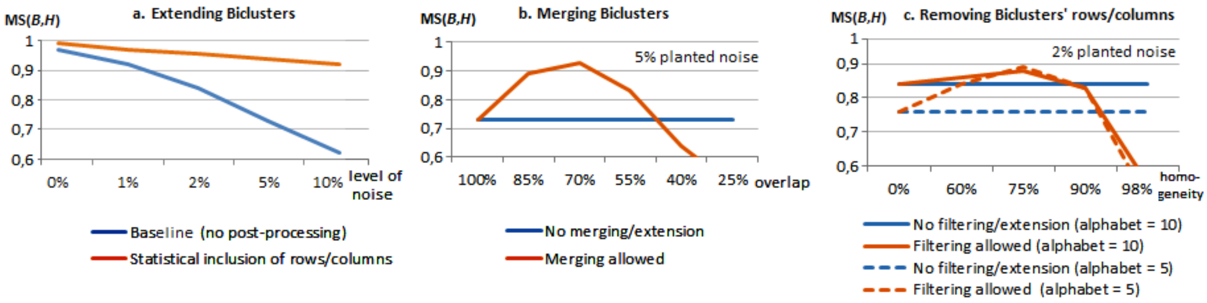

Figure S8: Impact of postprocessing options on the discovery of pattern-based biclusters from noisy data:
a. Impact of extending biclusters for data with varying percentage of noisy elements;
b. Impact of merging biclusters with varying overlapping degrees over data with 5% of noisy elements;
c. Impact of removing rows/columns in biclusters when considering varying homogeneity degrees (based on the mean squared residue [6]) and 2% of planted noise.

Missings. Finally, we generated data with a percentage of missing values ranging from 0 to 10% for the 1000×100 setting (that is, from 0 to 10.000 elements in the matrix). Since only few biclustering methods are able to deal with missing values, we used BicPAMS (parameterized with the Charm algorithm, 10 symbols and remaining default options) and measured the impact of using varying strategies to handle missing values (described in the main document). Note that 10% is already considered a very critical

number of missings that may compromise the ability to retrieve the true biclusters. As illustrated in Figure S9, we observe that this problem can be mitigated if biclustering methods are able to use robust strategies to deal with missing values. First, $MS(\mathcal{B},\mathcal{H})$ under the baseline strategy (remove missings) significantly decreases from 97% to near 70% when the percentage of missings reaches 10%. Second, the ability to retrieve the planted biclusters increases when considering the nearest 2-3 values against the strategies that consider the closest value only (simple imputation). In fact, when estimating more than one value for a missing, there is an increased chance to recover the original value and, therefore, of not damaging a planted bicluster. Although this solution can be supported in pattern-based biclustering by assigning multiple values to a single element, the majority of biclustering algorithms are not prepared to accommodate this behavior. Third, although inserting multiple values to replace a missing is an attractive option in terms of accuracy, its efficiency is penalized as the matrix becomes denser.

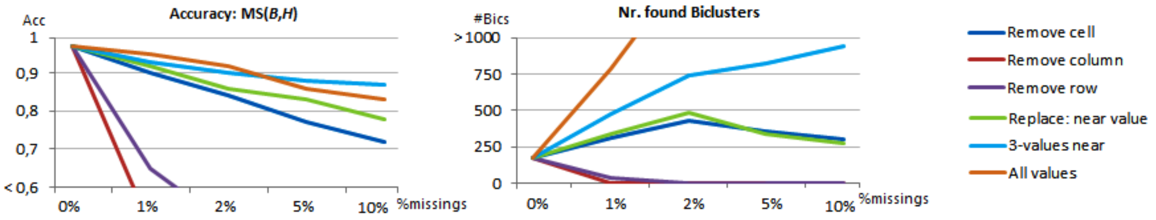

Figure S9: Comparing the impact of considering different strategies to handle missing values on the performance of pattern-based biclustering methods.

7 Performance in Sparse Data Contexts (Network Data)

Figure S10 compares the efficiency of BicNET [15] (made available in BicPAMS) with state-of-the-art biclustering algorithms with flexible coherence criteria using networks with varying size and density and planted modules with constant coherency. We selected FABIA [16], ISA [17], xMotifs [26], CC [6] and OPSM [3] to discover modules with flexible coherency. BicNET shows heightened efficiency levels. Understandably, as most of the remaining algorithms are only prepared to analyze (non-sparse) matrices, they show efficiency bottlenecks for even small networks. Furthermore, the majority is not able to accurately recover the planted modules as they cannot interpret missing interactions. Although SAMBA [39] and some pattern-based algorithms, such as BiMax and DECOB [8, 24], are able to discover dense modules efficiently, they are not prepared to discover modules with alternative coherence criteria.

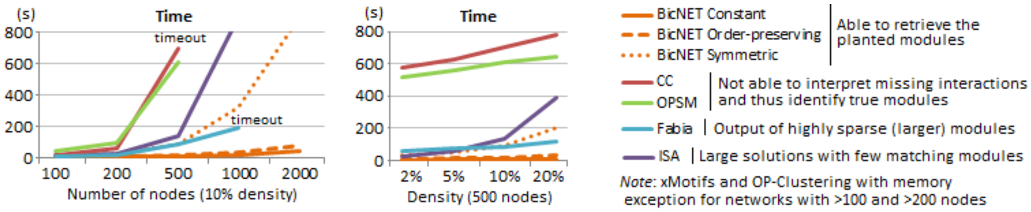

Figure S10: Efficiency of flexible biclustering algorithms to discover constant modules in synthetic networks with varying size and density.

Figure S11 compares the performance of BicNET with peer algorithms for discovering dense network modules (hypercliques) in the presence of noisy and missing interactions. This analysis clearly shows that existing searches for hypercliques have no tolerance to errors since their accuracy rapidly degrades for an increased number of planted noisy/missing interactions. Thus, they are not able to deal with the natural incompleteness and scoring uncertainty associated with biological networks. On the other hand, the observed accuracy levels of BicNET demonstrate its robustness to noise (validating the importance of assigning multiple ranges of weights for some interactions) and to missing interactions (showing the effectiveness of BicNET's postprocessing procedures).

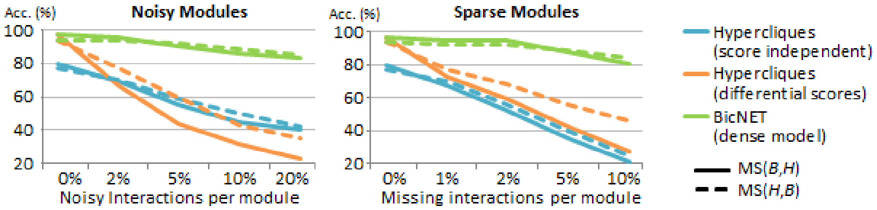

Figure S11: Accuracy of BicNET against searches aiming to discover hypercliques (i.e. perfectly dense modules) on networks (2000 nodes, 10% density) with varying degree of noise and missings.

8 Biological Relevance: Expression Data Analysis

To assess the biological relevance of BicPAMS solutions, we parameterize BicPAMS with multiple levels of expression ($|\mathcal{L}|\in\{4..7\}$), multiple coherences and distinct merging criteria (70% overlap; 55% overlap; and 90% overlap with extensions on rows that appear in another bicluster sharing a minimum 50% of the conditions/columns). We maintained the parameterizations of remaining approaches. The biological relevance of the biclusters was derived from assessing the over-representation Gene Ontology (GO) terms using an hypergeometric test. We consider a bicluster significant, when its genes (rows) show enrichment in one or more of the GO terms by having a (Bonferroni corrected) p-value below 0.01. In what follows, we analyze the: 1) functional enrichment and 2) transcriptional regulation of pattern-based biclusters. Part of the following results were previously reported in [11–13].

Functional Enrichment. Table S2 provides a compact view on the biological significance of the compared approaches. BicPAM is able to discover the largest number of (non-similar) biclusters with significantly enriched terms for each dataset. The analysis of these terms against the significant terms found in other biclustering solutions shows the completeness of BicPAM's solutions (as they cover the majority of the gathered biological functions per dataset), together with the exclusivity and relevance of BicPAM solutions (as they model biclusters with significantly enriched GO-terms that are not discovered by the remaining approaches). Although peer pattern-based solutions also find a large number of biclusters with significantly enriched terms, these terms have lower significance. This is due to the fact that these approaches do not provide noise-correction procedures to minimize the item-boundaries problem and cannot discover non-constant biclusters. Subsequent analyzes (Tables S3-S9) provide further empirical

evidence for the relevance, completeness and exclusivity of BicPAM solutions.

Table S3 shows the number of biologically significant biclusters found by BicPAM when recurring to different closing strategies. For this analysis, we varied the merging criteria (from a tight 95% overlapping threshold up to a relaxed 50% threshold) and provided the possibility to extend and reduce biclusters. A bicluster is considered to be highly significant if it has at least one enriched term with a corrected p-value below 0.01. To complement this analysis, Table S4 lists some of the most significant biological processes associated with these enriched terms for each data setting. Table S5 shows an illustrative set of the enriched pattern-based biclusters. Such biclusters could hardly be discovered by peer biclustering methods, since many of them include conditions with multiple degrees of expression (B1, B2 and B5)[11] and non-constant profiles (B8)[12]. All of these biclusters have heightened biological significance as observed by the number of highly enriched terms after Bonferroni correction. Interestingly, we also observe that different quality criteria can lead to biclusters with distinct shapes (B4 and B5).

Dataset	*Approach*	*♯Bics*	*Avg.♯Genes ×♯Conds*	*♯Bics sig. enriched*	*Coverage and exclusivity of enriched GO terms*
dlblc (human genome)	BicPAM	56	83×7	43 (77%)	Highest number of exclusively enriched terms (partial list in Table 4).
	BiModule	322	62×4	79 (25%)	Absence of closing options leads to redundant and less significant terms.
	DeBi	31	73×6	21 (68%)	Loss of relevant terms due to the inability to discover all maximal biclusters.
	CC	10	41×33	5 (50%)	Exclusive bicluster related with circulatory & cardiovascular system development.
	ISA	72	23×8	8 (11%)	Exclusive bicluster for extracellular structure organization and heparin binding.
	Plaid	3	12×49	1 (33%)	Majority of genes modeled in a single background bicluster with general terms.
	Fabia	10	79×35	6 (60%)	Small bicluster with superior enrichment of antigen binding functions.
	Bexpa	10	16×87	2 (20%)	Small sets of genes supported by large number of conditions.
	Samba	100	17×6	18 (18%)	Dedicated terms for antigen processing, peptide cross-linking and disassembly.
	OPSM	12	128×5	5 (42%)	High variance of ♯genes and ♯conditions; some of the biclusters with low ♯genes (coherency across high ♯conditions) have exclusive significantly enriched terms.
hughes (yeast genome)	BicPAM	47	360×7	38 (81%)	Exclusive enriched terms due to flexible coherency and post-processing criteria.
	BiModule	219	285×4	43 (20%)	Terms with lower sig. than terms from noise-tolerant BicPAM solutions.
	DeBi	28	317×7	21 (75%)	Terms observed across very small sets of conditions (≤5) are not enriched.
	CC	10	228×58	6 (60%)	GO terms covered by BicPAM constant biclusters.
	ISA	8	120×4	5 (63%)	Small biclusters with exclusive significance GO terms: spindle pole and karyogamy.
	Plaid	8	78×39	3 (38%)	One bicluster with higher significance for fungal-type cell wall assembly.
	Fabia	10	210×49	5 (50%)	Higher significance observed for actin cortical patch and oxidoreductase GO-terms.
	Bexpa	72	42×49	1 (10%)	Low number of enriched terms (probably due to the low ♯genes per bicluster).
	Samba	120	18×9	11 (9%)	Enriched terms covered by pattern-based biclustering solutions.
	OPSM	6	531×4	3 (50%)	Exclusive bicluster for the negative regulation of metabolic processes.
gasch (yeast genome)	BicPAM	149	411×8	123 (83%)	Large diversity of highly significant GO-terms (partial list in Table 4).
	BiModule	653	287×4	159 (24%)	Large but incomplete set of GO-terms as it excludes non-constant biclusters.
	DeBi	82	310×6	61 (74%)	Significance of terms slightly differ than BicPAM due to the handling of noise.
	CC	10	203×79	7 (70%)	Enriched terms appear in BicPAM solutions with higher significance.
	ISA	23	292×22	18 (78%)	Enriched terms covered by pattern-based biclustering solutions.
	Plaid	6	48×12	3 (50%)	Biclusters (apart from background layer) with lower enrichments than peers.
	Fabia	10	310×41	8 (80%)	Bicluster with higher sig. for specific proteasome complexes.
	Bexpa	10	63×29	3 (33%)	The few biclusters with deviation in size (higher ♯genes) are significant.
	OPSM	16	212×8	11 (69%)	One bicluster with higher significance for pre-ribosome functions.

Table S2: Comparing the biological relevance and novelty of different biclustering solutions.

[11]Illustrating, the bicluster identified as *B1*, with 83 genes from the human genome with coherent expression across 7 conditions, was discovered in *dlblc* using 6 levels of expression. These genes showed very low expression (A) on 2 samples, low expression (B) on 1 sample and very high expression (F) on 4 samples. Over 40 terms were highly enriched, with the top terms being related with immune defense responses and signaling functions associated to immunomodulating agents, an expected regulatory response to chemotherapy (yet differing from the biclusters identified by peer algorithms).

[12]The bicluster B8, found in *gasch* dataset, contains 521 genes from yeast genome with coherent expression across 10 time points from three different stress conditions (nitrogen depletion, heat stress and diauxic shift).

Dataset	Closing option	♯Bics	Avg. Area	♯Filtered bics	♯Highly sig. bics	♯Sig. bics
dlblc	merging	4803	81×7	28	22	5
	relaxed merging + reductions	980	83×9	24	19	3
	tight merging + extensions	7652	79×6	27	25	2
hughes	merge	6311	432×6	36	19	12
	relaxed merging + reductions	1259	492×7	22	12	8
	tight merging + extensions	9210	398×5	39	22	11
gasch	merge	27031	392×8	89	66	12
	relaxed merging + reductions	2177	486×11	67	49	11
	tight merging + extensions	52123	367×7	92	79	9

Table S3: Summary on the biological relevance of BicPAM's biclusters. Tight, default and relaxed merging procedures are, respectively, associated with 95%, 70% and 50% overlapping thresholds.

Dataset	ID	Terms	Bicluster with best p-value	♯Genes
dlblc	Dl1	Translation processes (including translational initiation and elongation)	4.49E-5	81
	Dl2	Transmembrane-related processes (including Golgi apparatus and MHC protein complex)	5.40E-5	83
	Dl3	Defense response; processes related with intra-cellular communication, including receptor activity	4.91-5	162
	Dl4	Innate immune responses, including response to interferon-gamma	1.06E-4	58
	Dl5	Cellular responses to chemical stimulus, including response to cytokine stimulus	0.001	60
	Dl6	Processes targeting the membrane-enclosed lumen associated with the cell cycle process	2.92E-12	81
	Dl7	Immune system processes	1.27E-4	52
hughes	H1	Mitochondrion organization and translation; mitochondrial matrix	2.70E-39	416
	H2	Processes concerning the cell periphery and sporulation; cell wall constituent and organization	1.73E-4	370
	H3	Ribonucleoprotein complex biogenesis	3.61E-30	426
	H4	Metabolic and biosynthetic processes of cellular amino acids and carboxylic acids	1.3E-25	581
	H5	Metabolic processes of organonitrogen and sulfur compounds	1.62E-4	504
gasch	G1	Cellular response to oxidative stress; generation of precursor metabolites and energy	2.37E-4	296
	G2	Processes to generate precursor metabolites and energy, including the tricarboxylic acid cycle	1.16E-14	954
	G3	Retrotransposon nucleocapsid; viral procapsid maturation	4.34E-6	102
	G4	Processes targeting the intracellular organelle lumen and nuclear lumen	1.17E-47	263
	G5	Nucleolus; ncRNA metabolic processes	1.03E-61	611
	G6	Intracellular non-membrane-bounded organelle; structural molecule activity	5.33E-76	293
	G7	Processes targeting the cytosolic part and, in particular, the ribosomal subunit	1.61E-88	460
	G8	Mitochondrion organization; mitochondrial part; biogenesis of certain protein complexes	2.06E-26	592
	G9	Regulation of cellular (macromolecule) biosynthetic processes; protein modification process	2.28E-13	1019
	G10	Organic substance catabolic and metabolic processes (including carbohydrates)	1.02E-15	648
	G11	General processes associated with ribonucleoprotein complex biogenesis	1.08E-94	784
	G12	Metabolic process of nitrogen compound and carboxylic/organic amino acids	1.84E-16	434
	G13	Binding processes targeting the nuclear part and preribosome	2.87E-11	508
	G14	Cellular processes involved in reproduction	0.001	435
	G15	Processes related with vacuolar transport; chromosome	5.09E-7	606

Table S4: Terms highly enriched in BicPAM's biclusters

In order to assess the importance of modeling plaid structures, we assessed solutions delivered by BiP with 10% noise-tolerant checks and in-between relaxation. This analysis is synthesized in Table S6 for $|\mathcal{L}|$=7 levels of expression. We can observe that the correct recovery of excluded elements from biclusters due to plaid effects leads to solutions with structural differences and a higher number of significantly enriched terms. Table S7 shows basic properties of BiP biclusters with terms only enriched under a plaid model. We can observe that such biclusters could hardly be discovered by existing plaid models provided by peer algorithms due to the flexibility associated with the allowed types of biclusters, alphabet length (number of expression levels) and target relaxation criteria. Finally, Table S8 provides illustrative sets of overlapping transcriptional modules found in the *dlblc* and *gasch* datasets together with the significantly enriched terms per module (bicluster). We used the modular decomposition of gene regulation activity

Dataset	ID	Pattern	Items $\mathcal{L}$	Closing Options	Type	♯Genes	♯Conds	♯p−values <0.01	♯p−values [0.01,0.05]	Best p-value
dlblc	B1	FAABFFF	A-F	Merging with tight overlapping	constant	83	7	41	21	1.97E-10
	B2	AAABCA	A-C	Extensions allowed (with tight merging)	constant	153	8	9	1	2.27E-12
	B3	AAA/../EEE	A-E	Reducing with high homogeneity	multiplicative	119	5	5	18	4.12E-8
hughes	B4	EEECEE	A-E	Merging allowed	constant	581	6	12	7	1.31E-25
	B5	CCDCBCBCC	A-E	Merging with relaxed overlapping	constant	654	10	16	4	1.31E-17
	B6	AAAAA/../G..G	A-G	Merging with tight overlapping	additive	476	6	12	10	1.92E-6
gasch	B7	AAAGGGA	A-G	Merging with tight overlapping	multiplicative	483	7	57	10	1.24E-81
	B8	AAABACCCAA	A-E	Merging allowed	additive	521	10	17	5	4.57E-12

Table S5: Illustrative set of biclusters with different properties and heightened biological relevance.

provided by the target plaid models. Complementarily, we used GOrilla and Yeastract tools to explore the hierarchy of the enriched terms per bicluster in order to identify shared parental terms (specialization relations) or cross-term interactions (dependency relations) between the enriched terms (analyzes reported in [11]). Illustrating, the set S_0 from *dlblc* contains 2 biclusters discovered using 6 expression levels (3 up-regulated and 3 down-regulated) showing moderate activation ($\forall_{(i,j)\in B_0 \oplus B_1} a_{ij} \in \{1,2\}$) yet high activation in the areas where they overlap ($\forall_{(i,j)\in B_0 \cap B_1} a_{ij} \in \{2,3,4\}$). Constrasting, the set S_1 defines three biclusters according to an is-part-of relation (B_2 and B_3 are contained in larger B_4) with baseline activity $a_{(i,j)\in B_2 \oplus B_3 \oplus B_4} \in \{2,3,4\}$ and activity in the overlapping areas $a_{(i,j)\in B_2 \cup B_4} \in \{3,4\}$.

Data	*Approach*	*Avg.♯Genes ×♯Conds*	*♯Sig.Bics (no plaid)*	*♯Sig.Bics (plaid)*
gasch	BiP In-between	392×7	54	61
gasch	BiP Approximated	378×7	54	58
gasch	BiP f-Multiplicative	383×6	54	56
dlbcl	BiP In-between	82×6	17	19
dlbcl	BiP f-Weighted	81×6	17	19

Table S6: Biological relevance of BiP models based on the number of biclusters with significantly enriched terms.

ID	*Data*	*|L|*	*Type*	*Composition*	*♯genes ×♯conds*	*♯terms p<0.01 of candidates*	*♯terms p<0.01 (plaid)*
B10	gasch	7	Additive	In-Between	559×6	51	58
B11	gasch	6	Constant	Approximated	482×9	5	6
B12	dlblc	5	Constant	In-Between	142×5	8	10
B13	dlblc	5	Additive	Approximated	118×5	5	11

Table S7: Illustrative biclusters with GO terms significantly enriched under a relaxed plaid assumption.

When applying BicSPAM to discover order-preserving biclusters, we were able to derive an average of 68 significant (and non-similar) biclusters with default parameterizations on these datasets (assuming a minimum number of δ=5 conditions). In particular, when considering symmetries, the average number of significant biclusters increases to over 80 biclusters with a larger number of elements in average. This is a critical observation since it means that there are groups of genes with biological relevance that can only be discovered through biclustering under a flexible order-preserving setting when symmetries are considered. Figure S12 illustrates the impact of including symmetries when mining the gasch dataset (with

Data	*Genome*	*Set ID*	*Biclusters*	*♯shared Genes*	*♯shared Conds*	*♯Levels Expression*	*Relaxation*
dlblc	human	S0	{B0,B1}	16	4	6	Approximate
		S1	{B2,B3,B4}	{54,59}	{3,3}	8	In-Between
gasch	yeast	S2	{B5,B6,B7}	{87,73,78}	{3,3,3}	7	In-Between
		S3	{B8}	–	–	7	Approximate
		S4	{B9,B10}	58	3	8	In-Between
		S5	{B11,B12}	132	4	8	f-Weighted

Bic ID	*Pattern* φ_B	*Overlapping expression*	*♯Genes*	*♯Terms* $<10^{-3}$	*Notes*
B0	U1U2U2U2	{U2,U3}	32	12	*dependency* interaction
B1	U2U2U1U1	{U2,U3}	39	7	
B2	U–U–U–	{U4}	54	27	*is-part-of* relation with B2⊆B4 and B3⊆B4
B3	U–U–U–	{U3,U4}	59	25	
B4	U2U3U3U4U3	{U3,U4}	81	33	
B5	U1U2U2U2	{U2,U3}	145	9	pairwise *dependency* interaction
B6	U2U2U2U2	{U2,U3}	218	17	
B7	U2U2U2U1	{U2,U3}	185	33	
B8	D1D1D1D1	{D2,D3}	191	22	*extended* bicluster
B9	D2D2D2D2	{D2,D3}	121	14	*dependency* interaction
B10	D2D2D2D2	{D2,D3}	131	26	
B11	D–D–D–	{D2,D3}	127	14	*is-part-of* relation with B11⊆B12
B12	D1D1D1D1D1	{D2,D3}	197	21	

Table S8: Properties of the 6 illustrative sets of meaningfully overlapping biclusters.

heat conditions only). Interestingly, we can see that order-preserving solutions that allow for symmetric behavior are able to capture a higher number of biclusters with larger sizes on average. Finally, Table S9 provides basic properties of an illustrative set of order-preserving biclusters with statistical significance.

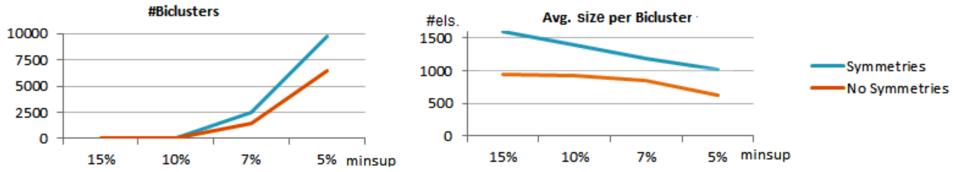

Figure S12: Relevance of order-preserving solutions with symmetries (yeast response to heat stress).

Dataset	♯Genes	♯Conds	♯Precedences	♯Items	Notes	♯p-values <0.01	♯p-values [0.01,0.05]	Best p-value
dlbcl	179	6	4	20	No closing options	5	2	3.12E-4
dlbcl	207	9	5	25	Merging allowed	6	1	2.33E-5
yeast	167	5	3	10	No closing options	11	3	2.12E-4
yeast	240	8	4	15	Extensions allowed	10	1	7.13E-7
colon	769	6	4	25	Merging allowed	12	2	6.08E-8
leukemia	1645	6	3	20	Extensions allowed	9	2	3.47E-9

Table S9: Illustrative biclusters passing the GO term-enrichment test at 1% and 5% significance levels.

Transcriptional Regulation. To complement the results on functional enrichment, we analyzed the enriched transcription factors (TFs) using the TFCONES database [20] (human genome) and Yeastract

database [40] (yeast genome) using a corrected hyper-geometric statistical test. An extended analysis of the TFs associated with the biclusters listed in Table S13 is now provided in Table S10. In this analysis we retrieved the more *representative* TFs – high coverage of the genes in the biclusters – and *significant* – high functional enrichment (p-value<1E-3). In line with the goal of *dlblc* and *gasch* experiments [9,32], we observe that the identified TFs are either directly or indirectly related with responses to chemotherapy (human) [20, 33] and stress conditions (yeast) [7, 40]. Consider the enriched TFs for the sample set of biclusters found in the *dlblc* dataset. Some of the TFs act as putative tumor suppressors include: ANP32A, LZTS1 (protein-coding silenced in rapidly metastasizing and metastatic tumor cells), RUNX3 (protein that binds to the core site of leukemia virus, also frequently silenced in cancer), HCLS1 (antigen receptor signaling deletion in lymphoid cells), IRF1 (protein that stimulates immune responses and regulates tumor cell differentiation), HIF1A (gene responsible for tumor angiogenesis and pathophysiology of ischemic disease), HDAC1 (complex interacting with retinoblastoma tumor-suppressor proteins), TCF3 (protein regulating lymphopoiesis as its deletion is associated with lymphoblastic and acute leukima malignancies) [20, 33]. Other TFs are dedicated to regulate cell proliferation include the STAT families, CREG1, MEF2B, ARID5B, and BCL3 [33]. Understandably, we also observed the B-cell lymphoma protein (BCL6 and its paralog coding gene BCL6B) and other leukemia-related disease genes involved in lymphoma pathogenes, such as BCL11A [20]. Complementarily, immune responses are associated with TRIM22 antiviral proteins, CEBPB, NFATC2 complex, and GTF2I for activating immunoglobulin heavy-chain transcription upon B-lymphocyte activation [33]. Finally, consider the subset of highly enriched TFs (regulating over 50% of the genes of each bicluster) provided in Table S10 for the sample set of biclusters found in *gasch* data. Although the enriched TFs regulate very distinct processes (see Table S13), most TFs are activated in stress conditions, namely: Yap1p, Cin5p and Hap2p during oxidative stress; Gcn4p, Msn2p and Msn4p during amino acid starvation; Hsf1p during variable heat shock ele-

Dataset	*Bic.ID* (Table S13)	*Highly enriched TFs*
dlblc	Dl1	BCL11A, LZTS1, GTF2I, HCLS1, HDAC1, MBD4, MEF2B, NCOA3, STAT6
	Dl2	ANP32A, HCLS1, IRF1, MNDA, NCOA1, RUNX3, STAT1, TRIM22, TRIP10
	Dl3	BCL3, TRIM22, ANP32A, ARID5B, CEBPB, CREG1, IRF1, PFDN5, STAT1
	Dl4	ANP32A, IRF1, NCOA1, STAT1, TRIM22
	Dl5	ANP32A, IRF1, NCOA1, STAT1, TRIM22
	Dl6	BCL6, BCL6B, HIF1A, ILF2, POU2AF1, SERTAD1, TCF3
	Dl7	CREG1, IRF1, TRIM22, ANP32A, STAT1
gasch	G1	Sfp1p, Msn2p, Msn4p, Cst6p, Abf1p, Sok2p, Bas1p
	G2	Sfp1p, Tec1p, Ste12p, Msn2p, Bas1p, Sok2p, Msn4p, Gcn4p
	G3	Snf6p, Tec1p, Ste12p, Rap1p, Sin4p, Abf1p, Snf2p, Ash1p
	G4	Sfp1p, Swi5p, Cst6p, Tup1p, Spt20p, Ash1p, Spt10p
	G5	Sfp1p, Ace2p, Cst6p, Tup1p, Msn2p, Spt10p, Spt20p
	G6	Hsf1p, Spt23p, Mga2p, Sfp1p, Spt10p, Msn2p, Gcr1p, Gcn4p
	G7	Sfp1p, Swi5p, Tup1p, Spt10p, Spt20p, Gcr1p, Sin3p, Mga2p
	G8	Yap1p, Ace2p, Sfp1p, Msn2p, Ash1p, Msn4p, Abf1p
	G9	Ace2p, Sfp1p, Tec1p, Ste12p, Ash1p, Bas1p, Gcn4p, Sok2p
	G10	Cin5p, Gcn4p, Msn4p, Sfp1p, Msn2p, Tec1p, Ste12p, Sok2p
	G11	Sfp1p, Ace2p, Cst6p, Snf6p, Rap1p, Tup1p, Spt10p, Swi5p
	G12	Gcn4p, Sfp1p, Ace2p, Tec1p, Ste12p, Ash1p
	G13	Snf6p, Tup1p, Snf2p, Cst6p, Sin4p, Rap1p, Swi3p, Hap2p
	G14	Yap1p, Tec1p, Msn2p, Msn4p, Ste12p, Sok2p
	G15	Sfp1p, Tec1p, Ste12p, Bas1p, Sok2p, Yrm1p

Table S10: Enriched TFs associated with putative modules given by BicPAMS biclusters from Table S13.

ments including hyperthermia; Sfp1p during DNA damage; and Spt23p and Mga2p during cooling [7]. The stress conditions are associated with invasive growth (regulated by Tec1p, Ste12p, Ash1p and Sok2p), and with the need for chromatin remodeling (regulated by Snf6p, Snf2p, Spt20p, Tup1p and Swi3p) and DNA repair (regulated for instance by Abf1p and Spt10p) [7, 40].

9 Biological Relevance: Network Data Analysis

Results gathered from the application of BicPAMS over real biological networks are provided in three parts. First, we show basic statistics that motivate the relevance of using BicPAMS against peer algorithms. Second, we explore the biological relevance of the retrieved modules when considering varying levels of tolerance to noise and different forms of coherency. Finally, we select an illustrative set of non-trivial modules (such as modules characterized by the presence of plaid effects, flexible constant patterns or symmetries), and provide a brief analysis of their enriched terms and transcription factors.

Figure S13 shows some of the properties of BicPAMS solutions for the selected four biological networks. In particular, all of the modules discovered in DRYGIN's yeast GIs and STRING's yeast GIs were significantly enriched. BicPAMS was able to discover the largest number of (non-similar and statistically significant) biclusters. The analysis of the enriched terms for these modules (see Tables S11 and S13) against the significant terms found in other biclustering solutions supports the completeness of BicPAMS solutions, as well as their exclusivity since the majority of the enriched modules were not discovered by peer algorithms. The biological significance of peer biclustering algorithms focused on dense regions is further hampered by noise and discretization errors (in accordance with Figure S13). Alternative biclustering algorithms able to discover non-dense regions were not able to scale. The subsequent analyzes (Tables S11–S14) provide further empirical evidence for the relevance, completeness and exclusivity of BicPAMS solutions.

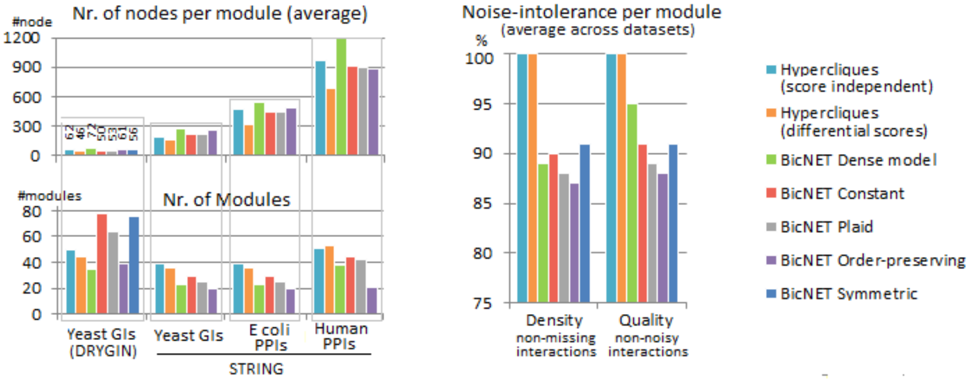

Figure S13: Properties of BicPAMS/BicNET solutions against hypercliques discovered in GI and PPI networks when considering varying coherency criteria.

Modules with Flexible Coherency. A subset of the overall modules collected from the application of

BicPAMS over the selected biological networks is provided in Table S11. This table gathers modules with varying: tolerance to noise (overlapping threshold for merging procedures varied between 60% and 90%), coherency assumption (dense, constant and order-preserving models) and coherency strength (D_1-D_4 with $\mathcal{L}$={-2,-1,1,2}, Y_1-Y_5 and H_1-H_3 with $\mathcal{L}$={1,2,3}, Y_6 and H_4 with $\mathcal{L}$={1,2,3,4}). All of the modules were discovered using multi-item assignments whenever values were found to be near a discretization boundary. The collected results show that all of BicPAMS modules had not only highly enriched terms, but also the enriched terms were found to be functionally related (taxonomically closed biological processes [23]). These results suggest that the discovered modules are characterized by a cohesive set of putative biological functions. Three major observations can be retrieved. First, the combination of the dense model with the provided procedures to foster robustness leads to higher enrichment factors as key genes/proteins with subtler yet functional relevance were not excluded from the modules. Nevertheless, this form of coherency is mainly associated with broader biological processes, such as general metabolic and regulatory processes (see Y_1, Y_2, H_1 and H_2 modules). Second, the constant model is indicated to guarantee a focus on less trivial modules associated with a compact set of more specific biological processes. Modules Y_3-Y_6, H_3-H_4 and D_2-D_4 are example of the relevance of considering non-dense interactions since these interactions are often related with latent or secondary (yet critical) cellular functions. Third, the order-preserving coherency is associated with modules as large as the ones provided under the noise-tolerant dense coherency, yet with the additional benefit of enabling the presence of weaker interactions as long as their coherency among the nodes is respected.

	ID	Homogeneity	♯Nodes $\|I\|\times\|J\|$	Putative functionality: group of enriched terms (p<1E-10)
STRING(yeast)	Y1	dense (high noise-tolerance)	231×14	Metabolic processes with incidence on protein, peptide and amide metabolism and biosynthesis.
	Y2	dense (medium noise-tolerance)	217×9	Metabolism of nitrogen compounds and some organic substances.
	Y3	constant (few high a_{ij})	103×8	Amino acid activation and tRNA metabolism for tRNA aminoacylation.
	Y4	constant (few high a_{ij})	206×6	Organic acid metabolic process and its subterms.
	Y5	constant (few high or low a_{ij})	55×7	Signal transduction and its subterms.
	Y6	constant (few high or low a_{ij})	43×6	Phosphorylation related terms (with incidence on protein phosphorylation).
	Y7	order-preserving	176×12	Transport of organic acids (with incidence on aminoacid transmembrane transport).
	Y8	order-preserving	235×9	Oxidation-reduction process and metabolism of aminoacids. Assembly of ribonucleoprotein.
	Y9	order-pres. (few high a_{ij})	146×8	Transport of molecules (highest enrichment found for drug transmembrane).
STR.(human)	H1	dense (high noise-tolerance)	811×28	Multiple metabolic processes with incidence on transcription activity.
	H2	dense (high noise-tolerance)	787×25	Regulation of metabolic processes (both positive and negative regulation).
	H3	constant (few high a_{ij})	693×14	Regulation of intracellular signal transduction (over 20 highly enriched terms).
	H4	constant (few high a_{ij})	645×10	Regulation of molecular functions (incidence on catalytic activity).
	H5	order-preserving	720×24	Establishment of protein localization (protein targeting to ER and membrane).
	H6	order-preserving	733×29	Protein phosphorylation and its subterms.
DryGIN	D1	dense (high noise-tolerance)	28×17	Organelle localization (establishment of spindle and nuclear localization).
	D2	constant (with pos&neg a_{ij})	22×10	Chromatin remodeling and nucleosome organization.
	D3	constant (with pos&neg a_{ij})	21×7	Transport processes for the establishment of protein localization.
	D4	constant (with pos&neg a_{ij})	19×9	Regulation of growth (incidence on filamentous growth).
	D5	order-preserving	39×7	Organelle and nucleous organization.
	D6	order-preserving	54×6	Regulation of cellular metabolic processes (both positive and negative regulation).

Table S11: Description of the biological role of an illustrative set of BicNET's modules with varying properties.

Non-trivial Modules. The provided modules in Table S11 already show flexible properties to surpass the limitations of the existing methods for network module discovery. Even so, BicPAMS can be used to further disclose less trivial modules, such as modules characterized by the presence of constant patterns with multiple symbols, symmetries and plaid effects. Table S12 shows an illustrative set of such modules

with significantly enriched terms. All of the illustrated modules show coherent patterns of interaction between nodes and have an average amount of 5 to 10% of missing interactions. This analysis reinforces that BicPAMS is well positioned to find modules with varying size, coherency and quality. Illustrating, the constant modules G_6 and G_7 have, respectively, 23 and 47 nodes and distinct quality, being G_7 more tolerant to noisy interactions. Understandably, the number of nodes per module is naturally affected by the size and sparsity of the target network. The discovered modules clearly show non-trivial yet meaningful correlations (as they include interactions with coherent yet non-differential scores), whose relevance is pinpointed by the number of highly enriched terms after correction.

	ID	Type	♯Nodes $\|I\|\times\|J\|$	Items	♯Terms p<1E-15	Notes
DryGIN	G1	constant	18×9	{-4,..,-1}	27	Module with coherent strong (-4) and soft (-1) negative interactions.
	G2	symmetric	4×9	{-3,..,3}	13	Varying levels of strong (mainly positive) interactions ({±3,±2}).
	G3	symmetric	5×6	{-2,-1,1,2}	12	Module with either all positive or negative interactions per "row"-node ({±1,±2}).
	G4	constant	7×5	{1,2}	12	Module with coherent strong (2) and soft (1) positive interactions.
	G5	symmetric	7×5	{-2,-1,1,2}	11	Module with either all positive or negative interactions per "row"-node ({±1,±2}).
	G6	order	14×11	{-3,..,3}	25	Preserved precedences and co-occurrences per "row"-node before postprocessing.
	G7	order	42×8	{-2,-1,1,2}	50	Noise-tolerant module with mostly preserved orderings per "row"-node.
STRING	S1	order	155×14	{1,2,3,4}	169	Preserved precedences and co-occurrences per "row"-node before postprocessing.
	S2	constant	80×18	{1,2,3}	98	Module with mostly of non-dense interactions ({1,2}).
	S3	constant	83×10	{1,2}	93	Module with non-dense positive interactions before postprocessing ({1}).
	S4	constant	50×20	{1,2,3}	70	Module with non-dense positive interactions ({1,2}) before postprocessing.
	S5	constant	45×31	{1,2,3}	76	Module with mostly dense interactions (scores in {2,3}).
	S6	constant	55×85	{1,2}	143	Module with mostly dense interactions ({2}).

Table S12: Exclusivity and relevance of BicPAMS solutions: properties of found modules.

Table S13 lists some of the enriched termsfor the modules in Table S12, showing their functional coherence and role to unravel putative biological processes. Interestingly, as illustrated in Table S14, some of the identified modules are part of an additive plaid model (with in-between condition [14]). Illustrating, modules G_6 and S_4 share, respectively, 21% and 42% of their interactions with modules G_7 and S_2 under a plaid assumption. Some properties of the two illustrative sets of overlapping modules are provided in Table S14. Without this assumption, only smaller modules (excluding key nodes) could be obtained, resulting in a lower enrichment of their terms.

The analysis of the enriched transcription factors (TFs) for each putative biological process in Table S13 further supports the previous functional enrichment analyzes. For this end, we retrieved the TFs that are more *representative* (high coverage of the genes in the module) and *significant* (high functional enrichment: p-value<1E-3). Illustrating, G_1 has diverse TFs regulating different families of histones, such as Jhd1p [40]; in G_4 we found regulators of meiosis, including Sin3p [40]; the TFs of G_7 activate genes required for cytokinesis (exit from mitosis); in S_1 we found TFs associated with responses to oxygen-related stress, such as the activation of beta-oxidation genes by Pip2p [40]; proteins regulating S_2 respond to DNA damaging, such as Plm2p and Abf1p [7]; membrane sensors, such as Ure2p, are active in the regulation of genes in S_3; S_4 has proteins promoting the organization and remodeling of chromatin, including Abf1p, Plm2p and Rsc1p [7]; regulators of ribosomal biogenesis, such as Sfp1p (100% representativity), and of its subunits, such as Cse2p [40], are core TFs for S_6.

	ID	Terms description (♯)	♯Terms p<1E-15	♯Nodes
DryGIN	G1	Histone modification; regulation of histone H3-K79 methylation, histone H2B ubiquitination, H2B conserved C-terminal lysine ubiquitination, H3-K4 methylation (4);	6	27
	G2	Regulation of gluconeogenesis; glutamate metabolic and catabolic processes (2); nicotinamide riboside metabolic process; nicotinamide nucleotide biosynthetic process;	6	13
	G3	Positive and negative regulation of transcription from RNA polymerase II; Invasive growth response to glucose limitation and hyperosmotic salinity response by regulating RNA polymerase II (5);	5	12
	G4	Meiotic anaphase I; activation of anaphase-promoting complex activity involved in meiotic cell cycle;	4	12
	G5	Negative reg. of phospholipid biosynthesis; lipid homeostasis; isopropylmalate and oxaloacetate transport;	4	11
	G6	Cotranslational protein targeting to membrane; protein insertion into mitochondrial membrane; protein import into peroxisome membrane; reg. sporulation; actin filament bundle assembly involved in cytokinesis;	5	25
	G7	Acetate fermentation, acetyl-CoA biosynthesis (from acetate), reg. transcription on exit from mitosis;	7	50
STRING	S1	Response to hypoxia; oxidation-dependent protein catabolic process; anaerobic respiration; age-dependent response to reactive oxygen species; cellular response to oxidative stress;	36	169
	S2	Positive & negative reg. of mitotic and nuclear cell cycle, DNA replication, budding cell apical bud growth;	16	98
	S3	Transport of aerobic electron, acetyl-CoA, vacuolar transmembrane, amine, transport (5); ribose phosphate metabolic process; D-ribose metabolic and catabolic processes (2);	22	93
	S4	Heterochromatin maintenance involved in chromatin silencing; sister chromatid segregation;	6	70
	S5	Cytoplasmic and mitochondrial translation (4); regulation of translational fidelity; ADP biosynthesis;	6	76
	S6	rRNA processing; separation, cleavage & maturation of SSU-rRNA (5); ribosomal (large subunit) biogenesis;	14	143

Table S13: Illustrative set of biologically significant BicPAMS modules: description of the highly enriched terms in the modules presented in Table S12 [7, 40].

ID	Modules with meaningful overlapping regions	Pattern	♯Nodes $\|I\|\times\|J\|$	%Overlapping interactions
G6	G7 from Table S13 (orders preserved in overlapping regions before cumulative effect);	order	42×8	21%
	G8: tRNA re-export from nucleus; nuclear mRNA surveillance of mRNP export;	constant	12×10	62%
	G9: More general module (background) including cellular responses to pH;	constant	41×6	16%
S4	S2 from Table S13 (satisfying the relaxed additive model proposed in [11]);	constant	80×18	42%
	S7: Telomere maintenance; translocation; protein import into nucleous;	constant	104×20	37%
	S8: Response to ionizing radiation; ribose phosphate metabolic process;	constant	59×31	45%
	S9: Positive regulation of mitochondrial translation in response to stress;	constant	50×20	89%

Table S14: Sets of modules with meaningful overlapping areas (satisfying the in-between plaid assumption [11]).

Ending Note. When analyzing networks derived from knowledge-based repositories and literature (such as the networks from STRING [38]), flexible coherency and noise-robustness are critical to deal with uncertainty and with regions in the network where scores may be affected by research biases. When analyzing networks derived from data experiments (such as the GIs from DRYGIN [18]), the discovery of modules with non-necessarily strong interactions (e.g. given by the constant model) is critical to model less-predominant (yet key) biological processes, such as the ones associated with early stages of stimulation or disease.

10 Concluding Remarks

The gathered results support the relevance of BicPAMS to integrate state-of-the-art contributions from pattern-based biclustering. Results over synthetic data confirm the superiority of BicPAMS algorithms against peers, and their inherent ability to provide exhaustive yet efficient searches for biclusters with parameterizable coherency and quality. In particular, we demonstrate their ability to deal with varying

coherency criteria and structures of biclusters, to learn from sparse data, and to robustly handle arbitrary-high amounts of noisy and missing elements. Biological analyzes from the application of the algorithms in BicPAMS over gene expression data and biological networks reveal their unique ability to find complete, meaningful and non-trivial biclusters with heightened biological relevance that could not be discovered by other biclustering algorithms.

References

1. I. Assent, R. Krieger, E. Muller, and T. Seidl. DUSC: Dimensionality Unbiased Subspace Clustering. In *ICDM*, 2007.
2. Simon Barkow, Stefan Bleuler, Amela Prelić, Philip Zimmermann, and Eckart Zitzler. Bicat: a biclustering analysis toolbox. *Bioinformatics*, 22(10):1282–1283, May 2006.
3. Amir Ben-Dor, Benny Chor, Richard Karp, and Zohar Yakhini. Discovering local structure in gene expression data: the order-preserving submatrix problem. In *RECOMB*, pages 49–57, New York, NY, USA, 2002. ACM.
4. Doruk Bozdağ, Ashwin S. Kumar, and Umit V. Catalyurek. Comparative analysis of biclustering algorithms. In *BCB*, pages 265–274, New York, NY, USA, 2010. ACM.
5. J. Caldas and S. Kaski. Bayesian biclustering with the plaid model. In *IEEE Workshop on MLSP*, pages 291–296, Oct 2008.
6. Yizong Cheng and George M. Church. Biclustering of expression data. In *Intelligent Systems for Molecular Biology*, pages 93–103. AAAI Press, 2000.
7. J Michael Cherry, Eurie L Hong, Craig Amundsen, Rama Balakrishnan, Gail Binkley, Esther T Chan, Karen R Christie, Maria C Costanzo, Selina S Dwight, Stacia R Engel, DG Fisk, JE Hirschman, BC Hitz, K Karra, CJ Krieger, SR Miyasato, RS Nash, J Park, MS Skrzypek, M Simison, S Weng, and ED Wong. Saccharomyces genome database: the genomics resource of budding yeast. *Nucleic acids research*, page gkr1029, 2011.
8. Recep Colak, Flavia Moser, Jeffrey Shih-Chieh Chu, Alexander Schönhuth, Nansheng Chen, and Martin Ester. Module discovery by exhaustive search for densely connected, co-expressed regions in biomolecular interaction networks. *PLoS One*, 5(10):e13348, 2010.
9. Audrey P. Gasch, Paul T. Spellman, Camilla M. Kao, Orna Carmel-Harel, Michael B. Eisen, Gisela Storz, David Botstein, and Patrick O. Brown. Genomic expression programs in the response of yeast cells to environmental changes. *Molecular Biology of the Cell*, 11(12):4241–4257, 2000.
10. Jiajun Gu and Jun Liu. Bayesian biclustering of gene expression data. *BMC Genomics*, 9(Suppl 1):S4, 2008.
11. R. Henriques and S. Madeira. Biclustering with flexible plaid models to unravel interactions between biological processes. *Computational Biology and Bioinformatics, IEEE/ACM Transactions on*, 2015.
12. Rui Henriques and Sara Madeira. Bicpam: Pattern-based biclustering for biomedical data analysis. *Algorithms for Molecular Biology*, 9(1):27, 2014.
13. Rui Henriques and Sara Madeira. Bicspam: Flexible biclustering using sequential patterns. *BMC Bioinformatics*, 15:130, 2014.
14. Rui Henriques and Sara C. Madeira. Bip: Effective discovery of overlapping biclusters using flexible plaid models. In *BIOKDD, ACM SIGKDD*. ACM, 2014.
15. Rui Henriques and Sara C. Madeira. Bicnet: Efficient biclustering of biological networks to unravel non-trivial modules. In *Algorithms in Bioinformatics (WABI)*, LNCS. Springer-Verlag, 2015.
16. Sepp Hochreiter, Ulrich Bodenhofer, Martin Heusel, Andreas Mayr, Andreas Mitterecker, Adetayo Kasim, Tatsiana Khamiakova, Suzy Van Sanden, Dan Lin, Willem Talloen, Luc Bijnens, Hinrich W. H. Göhlmann, Ziv Shkedy, and Djork-Arné Clevert. FABIA: factor analysis for bicluster acquisition. *Bioinformatics*, 26(12):1520–1527, June 2010.
17. Jan Ihmels, Sven Bergmann, and Naama Barkai. Defining transcription modules using large-scale gene expression data. *Bioinformatics*, 20(13):1993–2003, September 2004.
18. Judice L. Y. Koh, Huiming Ding, Michael Costanzo, Anastasia Baryshnikova, Kiana Toufighi, Gary D. Bader, Chad L. Myers, Brenda J. Andrews, and Charles Boone. Drygin: a database of quantitative genetic interaction networks in yeast. *Nucleic Acids Research*, 38(suppl 1):D502–D507, 2010.
19. Laura Lazzeroni and Art Owen. Plaid models for gene expression data. *Statistica Sinica*, 12:61–86, 2000.
20. Alison P Lee, Yuchen Yang, Sydney Brenner, and Byrappa Venkatesh. Tfcones: a database of vertebrate transcription factor-encoding genes and their associated conserved noncoding elements. *BMC genomics*, 8(1):441, 2007.

21. William Lee, Desiree Tillo, Nicolas Bray, Randall H. Morse, Ronald W. Davis, Timothy R. Hughes, and Corey Nislow. A high-resolution atlas of nucleosome occupancy in yeast. *Nature Genetics*, 39(10):1235–1244, September 2007.

22. Jinze Liu and Wei Wang. Op-cluster: Clustering by tendency in high dimensional space. In *ICDM*, pages 187–, Washington, DC, USA, 2003. IEEE Computer Society.

23. David Martin, Christine Brun, Elisabeth Remy, Pierre Mouren, Denis Thieffry, and Bernard Jacq. Gotoolbox: functional analysis of gene datasets based on gene ontology. *Genome Biology*, (12):101, 2004.

24. Anirban Mukhopadhyay, Ujjwal Maulik, and Sanghamitra Bandyopadhyay. A novel biclustering approach to association rule mining for predicting hiv-1–human protein interactions. *PLoS ONE*, 7(4):e32289, 04 2012.

25. James Munkres. Algorithms for the Assignment and Transportation Problems. *Society for Ind. and Applied Math.*, 5(1):32–38, 1957.

26. T. M. Murali and Simon Kasif. Extracting conserved gene expression motifs from gene expression data. In *Pacific Symposium on Biocomputing*, pages 77–88, 2003.

27. Yoshifumi Okada, Wataru Fujibuchi, and Paul Horton. A biclustering method for gene expression module discovery using closed itemset enumeration algorithm. *IPSJ Transactions on Bioinformatics*, 48(SIG5):39–48, 2007.

28. Gaurav Pandey, Gowtham Atluri, Michael Steinbach, Chad L. Myers, and Vipin Kumar. An association analysis approach to biclustering. In *KDD*, pages 677–686, New York, NY, USA, 2009. ACM.

29. Anne Patrikainen and Marina Meila. Comparing subspace clusterings. *IEEE TKDE*, 18(7):902–916, July 2006.

30. Beatriz Pontes, Raúl Giráldez, and Jesús S Aguilar-Ruiz. Configurable pattern-based evolutionary biclustering of gene expression data. *Algorithms for Molecular Biology*, 8(1):4, 2013.

31. Amela Prelić, Stefan Bleuler, Philip Zimmermann, Anja Wille, Peter Bühlmann, Wilhelm Gruissem, Lars Hennig, Lothar Thiele, and Eckart Zitzler. A systematic comparison and evaluation of biclustering methods for gene expression data. *Bioinf.*, 22(9):1122–1129, 2006.

32. Andreas Rosenwald and *dlblc team*. The use of molecular profiling to predict survival after chemotherapy for diffuse large-B-cell lymphoma. *N Engl J Med*, 346(25):1937–1947, 2002.

33. Marilyn Safran, Irina Dalah, Justin Alexander, Naomi Rosen, Tsippi Iny Stein, Michael Shmoish, Noam Nativ, Iris Bahir, Tirza Doniger, Hagit Krug, A Sirota-Madi, T Olender, Y Golan, G Stelzer, A Harel, and D Lancet. Genecards version 3: the human gene integrator. *Database*, 2010:baq020, 2010.

34. Rodrigo Santamaria. Bcplaid: The plaid model bicluster algorithm, package 'biclust' https://cran.r-project.org/web/packages/biclust/biclust.pdf. pages 5–6, 2015.

35. Eran Segal, Alexis Battle, and Daphne Koller. Decomposing gene expression into cellular processes. In *Pacific Symp. on Biocomputing*, volume 8, pages 89–100, 2003.

36. Karlton Sequeira and Mohammed Zaki. Schism: a new approach to interesting subspace mining. *Int. J. Bus. Intell. Data Min.*, 1(2):137–160, December 2005.

37. Akdes Serin and Martin Vingron. Debi: Discovering differentially expressed biclusters using a frequent itemset approach. *Algorithms for Molecular Biology*, 6:1–12, 2011.

38. Damian Szklarczyk, Andrea Franceschini, Stefan Wyder, Kristoffer Forslund, Davide Heller, Jaime Huerta-Cepas, Milan Simonovic, Alexander Roth, Alberto Santos, Kalliopi P Tsafou, et al. String v10: protein–protein interaction networks, integrated over the tree of life. *Nucleic acids research*, page gku1003, 2014.

39. Amos Tanay, Roded Sharan, and Ron Shamir. Discovering statistically significant biclusters in gene expression data. *Bioinformatics*, 18:136–144, 2002.

40. M.C. Teixeira, P.T. Monteiro, J. Guerreiro, J.P. Gonçalves, N.P. Mira, S.C. dos Santos, T.R. Cabrito, M. Palma, C. Costa, A.P. Francisco, S.C. Madeira, A.L. Oliveira, A.T. Freitas, and I. Sá-Correia. The yeastract database: an upgraded information system for the analysis of gene and genomic transcription regulation in *Saccharomyces cerevisiae*. *Nucleic Acids Research*, (Database issue), 2014.

41. Heather Turner, Trevor Bailey, and Wojtek Krzanowski. Improved biclustering of microarray data demonstrated through systematic performance tests. *Comp. Stat. & Data Analysis*, 48(2):235–254, 2003.
